# Discovery of an AKT1-targeting compound from a traditional herbal formula for alcoholic liver disease via integrative computational and experimental approaches

**DOI:** 10.1186/s13020-025-01205-y

**Published:** 2025-10-07

**Authors:** Shuxuan Yang, Caiting Zou, Dexian Li, Jingxin Lin, Qinghong Chen, Meilin Chen, Chuanghai Wu, Andrew Hung, Yanyan Liu, Xiaomin Sun, Hong Li, Qi Wang, Xiaoshan Zhao

**Affiliations:** 1 School of Traditional Chinese Medicine, Southern Medical University, No.1023 South Shatai Road, Baiyun District, Guangzhou, 510515 Guangdong China; 2 School of Science, STEM College, RMIT University, Melbourne, VIC 3000 Australia; 3 Department of Traditional Chinese Medicine, National Institute of Traditional Chinese Medicine Constitution and Preventive Treatment, Beijing University of Chinese Medicine, Beijing, 100029 China; 4 Department of Traditional Chinese Medicine, Nanfang Hospital, Southern Medical University, No.1023 South Shatai Road, Baiyun District, Guangzhou, 510515 Guangdong China

**Keywords:** Traditional herbal medicine, Metabolomic profiling, Molecular docking, Natural product therapy, Computer-aided drug design, In silico

## Abstract

**Background:**

Alcoholic liver disease (ALD) poses a major global health challenge, with limited effective interventions. The Dampness-Heat Regulating Formula (DRF), a traditional Chinese herbal tea composed of nine edible medicinal herbs, has shown promise in mitigating alcohol-induced liver injury. This study aimed to identify its core active components and elucidate underlying mechanisms.

**Methods:**

Active compounds were retrieved from multiple databases and screened using chemical similarity, target prediction, and ADMET filtering. Disease-related targets were identified through public transcriptomic datasets. Three machine learning algorithms—random forest, support vector machine, and LASSO—were used to prioritize therapeutic targets. High-throughput molecular docking and virtual screening were combined with untargeted metabolomics to identify candidate compounds. The interaction between oleanolic acid (OA) and AKT1 was further verified by cellular thermal shift assay (CESTA). In vitro and in vivo assays were conducted to validate hepatoprotective effects. Additionally, the content of OA in DRF was quantified by HPLC to assess the relevance of experimental dosing.

**Results:**

A total of 690 candidate compounds and 33 ALD-associated targets were identified. AKT1 emerged as the top-ranked hub target. OA showed strong binding affinity to AKT1, and CESTA confirmed their direct interaction. Functional assays demonstrated that OA alleviated ethanol-induced damage in hepatocytes and zebrafish models. HPLC analysis confirmed that DRF contained physiologically relevant concentrations of OA, supporting the translational relevance of the selected doses.

**Conclusion:**

This study reveals a potential AKT1-centered mechanism through which DRF protects against ALD and identifies oleanolic acid as a bioactive compound with dual computational and experimental validation. It offers a scientific basis for integrating traditional herbal formulas with modern drug discovery approaches in the prevention of alcohol-related liver injury.

**Supplementary Information:**

The online version contains supplementary material available at 10.1186/s13020-025-01205-y.

## Introduction

Alcohol misuse is recognized as a major risk factor for disease, disability, and mortality worldwide [1]. Approximately 43% of the global population currently engages in alcohol consumption [2]. While age-standardized rates of heavy drinking are highest in European countries, the absolute burden may be even more severe in Asia [3]. Alcohol consumption significantly increases the risks of liver-related mortality, with alcoholic liver disease (ALD) raising the Liver disease mortality risk by 260-fold, cardiovascular mortality by 3.2-fold, and cancer mortality by 5.1-fold [1]. ALD is a chronic, progressive condition primarily caused by alcohol use disorder [4] or prolonged heavy drinking [5]. The early stages of ALD are often asymptomatic, especially in patients with steatosis or alcoholic steatohepatitis. However, as the disease progresses, approximately 10% to 20% of patients with ALD will progress to cirrhosis [6]. Patients with alcoholic hepatitis, who exhibit the three pathological features of steatosis, alcoholic hepatitis, and fibrosis, are at the highest risk of progressing to cirrhosis. Globally, approximately 3% of the population is affected by ALD, and the five-year mortality rate for patients with ALD exceeds 50% [7]. Additionally, the Coronavirus Disease 2019 pandemic has significantly increased the incidence and mortality of ALD. Patients with alcohol-related cirrhosis have a mortality rate twice that of those with cirrhosis caused by other factors, further exacerbating the global burden of ALD. All these factors are closely linked to high short-term mortality rates and result in substantial treatment and management costs [8, 9], posing a major challenge to global public health and economic development.

The onset of ALD is complex and multifactorial, involving oxidative stress driven by alcohol metabolism and reactive oxygen species, cellular stress, endoplasmic reticulum stress, gut-derived microbial factors, and epigenetic changes [6]. Current treatments, such as cortisol therapy and emerging drugs like metoprolol, interleukin-22 analogs, and interleukin-1β antagonists, are initiated only after liver damage occurs [10]. However, these treatment options have limited efficacy, particularly in patients with decompensated alcoholic cirrhosis, and are often associated with significant side effects. For instance, cortisol therapy, while effective in reducing inflammation, may increase the risk of infections and metabolic disorders, compromising patient outcomes [11], while metoprolol can cause bradycardia and fatigue [12]. When pharmacological treatments fail, Liver transplantation becomes the last resort. However, its accessibility is Limited, with only 54.4% of patients receiving a graft within one Year and a mortality rate of 12.2 deaths per 100 waiting list years [10]. Therefore, there is an urgent need for effective, low-side-effect therapies for early intervention and daily liver health management, such as dietary or functional food-based approaches, to prevent ALD progression and improve outcomes.

In recent years, Traditional Chinese Medicine (TCM) and natural products have gained widespread attention globally due to their demonstrated efficacy in improving liver function and reducing inflammation, with a lower incidence of adverse effects compared to conventional therapies [13, 14]. Compared to conventional therapies, TCM’s holistic approach targets multiple pathological mechanisms, such as oxidative stress, inflammation, and gut-liver axis dysregulation, addressing the root causes of ALD and offering a safer and more feasible treatment option [13]. The advantage of TCM formulas lies in their ability to tackle complex conditions through the synergistic effects of multiple herbs. For example, the combination of *Lonicera japonica* and *Taraxaci herba* has been shown to enhance anti-inflammatory and antioxidant effects in ALD. Therefore, the use of TCM compound therapy for ALD represents a promising therapeutic strategy, particularly due to its multi-target effects, individualized treatment approach, and potential for early intervention.

Dampness-Heat Regulating Formula (DRF) is a traditional Chinese herbal tea comprising nine medicinal herbs, all of which are classified as ‘medicine-food homology’ substances, meaning they are both medicinal and edible. These herbs include *Pogostemon cablin* (Huo xiang, HX), *Myristica fragrans* (Rou dou kou, RDK), *Coicis semen* (Yi yi ren, YYR), *Lonicera japonica* (Jin yin hua, JYH), *Portulaca oleracea* (Ma chi xian, MCX), *Mentha haplocalyx* (Bo he, BH), *Phaseoli semen* (Chi xiao dou, CXD), *Lophatheri herba* (Dan zhu ye, DZY), and *Taraxaci herba* (Pu gong ying, PGY). The combination of these herbs leverages their synergistic effects to regulate dampness-heat conditions, making DRF a promising candidate for managing ALD. Some of these plants have demonstrated anti-fibrotic or anti-inflammatory effects on the liver, as well as hepatoprotective properties [15–19]. However, the mechanisms underlying the combined effects of multiple herbs in DRF and the identification of its key active components remain unclear. Further research is needed to elucidate how these ingredients interact synergistically to exert their therapeutic benefits, particularly in the context of liver protection and disease modulation. Through a comprehensive systems-based investigation, we elucidated the therapeutic mechanisms and active components of DRF against ALD by integrating computational target prediction, transcriptomic profiling with machine learning analysis, metabolomic characterization, and experimental validation. This work provides a scientific foundation for developing evidence-based ALD therapeutics from TCM.

## Materials and methods

### In silico analysis

#### Computational compound-target mapping and functional relevance analysis

In this section, we performed a computational workflow integrating multi-database compound retrieval, target prediction, and functional annotation to uncover potential mechanistic links between DRF constituents and ALD.

##### Identification of DRF compounds

The compounds in DRF were retrieved from the Traditional Chinese Medicine Systems Pharmacology Database (TCMSP, https://tcmsp-e.com/), a widely used platform for network pharmacology studies on Chinese herbal formulas [20]. TCMSP contains data on 499 herbs from the *Chinese Pharmacopoeia*, encompassing 13,144 compounds with annotated pharmacokinetic properties [21]. All entries are manually curated and regularly updated. In this study, we searched the pinyin names of the nine DRF herbs to obtain compound structures and pharmacokinetic data. For compounds not found in TCMSP, supplementary information was collected from the BATMAN-TCM database (http://bionet.ncpsb.org.cn/batman-tcm/), a bioinformatics tool developed in 2016 for exploring TCM molecular mechanism [21].

##### Acquisition of potential targets for ALD

In this study, we adopted a three-step strategy to identify potential protein targets of DRF against ALD. First, compound-related targets were retrieved from TCMSP and BATMAN-TCM [22]. Second, inverse docking was performed via SwissTargetPrediction (http://swisstargetprediction.ch/), which predicts targets based on 2D and 3D similarity; targets with a probability > 0.05 were retained [23]. These were considered the most likely DRF-related targets. Then, ALD-associated targets were obtained from Open Targets [24] (https://platform.opentargets.org/), GeneCards [25] (https://www.genecards.org/), and Comparative Toxicogenomics Database (CTD) [26](http://ctdbase.org/), to discern common disease targets associated with ALD. All the gathered targets were standardized to official gene names using the UniProt database [27] (https://www.uniprot.org/). Finally, an online bioinformatics utility (http://www.bioinformatics.com.cn/) was employed to craft a Venn diagram, elucidating the shared targets between DRF and ALD.

#### Establishment of protein–protein interaction network

To systematically dissect the biological functions and interactions of the candidate targets, we employed the STRING database (https://string-db.org) to delineate a Protein–Protein Interaction (PPI) network [28]. We focused on targets identified as ‘*Homo sapiens*’. The connections between targets, shown by edge thickness, were based on ‘confidence’ scores, with a minimum threshold of ‘medium confidence (0.400)’. Only proteins with interactions were advanced for further analysis, while those without any connections were excluded.

#### Systems-level exploration of DRF compounds’ mechanisms in ALD

To explore the molecular mechanisms of DRF compounds in ALD, we conducted KEGG and GO enrichment analyses using the DAVID database [29], a tool for functional annotation of gene lists. Target genes (official symbols) were submitted with ‘*Homo sapiens*’ specified to ensure human-specific, biologically relevant results. KEGG enrichment analysis identified key signaling pathways potentially modulated by DRF compounds, many of which are implicated in disease progression [30]. The top 10 pathways (ranked by *p*-value) were visualized using Sankey diagrams generated via an online tool (http://www.bioinformatics.com.cn/), clearly illustrating pathway–gene associations [29].GO enrichment analysis further classified DRF-related genes into three domains: biological process, molecular function, and cellular component [31]. This provided detailed insights into the functional roles of these genes and their relevance to ALD.

#### Supercomputing-assisted docking analysis of DRF bioactives targeting ALD

To predict the molecular interactions between DRF-derived compounds and ALD-related protein targets, molecular docking simulations were conducted using the Sunway TaihuLight supercomputer at the National Supercomputing Center in Wuxi, China. All docking tasks were executed on nodes equipped with Shenwei 26,010 multi-core processors.

Chemical structures of all candidate ligands from DRF were retrieved from the PubChem database using their respective compound identifiers (CIDs). SMILES sequences and 3D SDF files were downloaded when available. For compounds lacking SDF formats, MOL2 structures from the TCMSP or 2D formats were used and subsequently converted to PDB format via Discovery Studio Visualizer 2024. Target protein structures were sourced from the AlphaFold Protein Structure Database (https://alphafold.com/). Missing fragments, if any, were reconstructed using SWISS-MODEL [32], and all protein files were uniformly preprocessed using Discovery Studio Visualizer 2024 to ensure compatibility for docking analysis.

Ligand-target docking simulations were performed using AutoDock Vina (v1.1.2) within the PyRx (v0.8) environment [33]. Protein and ligand structures were prepared and converted to PDBQT format, and ‘blind docking’ was conducted by generating maximized search boxes to cover the entire surface of each receptor [34]. All ligands were treated as fully flexible, while protein targets were kept rigid [35]. The docking exhaustiveness was set to 8 for all simulations. Binding affinity values were used to assess interaction strength, with thresholds of − 7.0 kcal/mol or lower considered indicative of strong binding, as supported by previous studies [22, 36]. Docking results were further evaluated based on binding energy rankings and visual inspection of receptor-ligand interactions.

#### Compound-target network visualization

To investigate interactions between DRF compounds and ALD-related targets, a compound-target network was built using compounds with binding affinity ≤ − 8.0 kcal/mol and ranked in the top 10 for each protein. This dual-filter ensured both strong binding and target specificity. The network was visualized in Cytoscape (v3.10.3, macOS), with node sizes scaled by degree centrality to highlight key hub molecules involved in DRF’s polypharmacological effects.

#### Structural cluster analysis and classification of compounds

To examine the Link between compound structures and bioactivity, we analyzed the physicochemical properties of 690 DRF compounds using OSIRIS DataWarrior (v5.5.0, macOS) [37] and ClassyFire (http://classyfire.wishartlab.com/) [38]. SMILES strings and docking energies were imported into DataWarrior for clustering based on structural similarity and binding affinity. Clusters with more than 7 compounds were further classified using ClassyFire, a robust automated taxonomy platform for chemical classification.

#### Structural clustering and chemotaxonomic classification of DRF compounds

The failure of drug development is often linked to poor pharmacokinetics and toxicity profiles of candidate compounds [39], making early ADMET (absorption, distribution, metabolism, excretion, and toxicity) evaluation essential. We used ADMETLAB 3.0 (https://admetlab3.scbdd.com/server/screening) to virtually screen DRF compounds based on pharmacological and toxicity parameters. Drug-likeness was assessed by PAINS alerts and Lipinski’s Rule violations [40], while oral bioavailability was evaluated via human intestinal absorption (HIA), and toxicity was predicted by Ames test and human hepatotoxicity (H-HT). Screening criteria for DRF compounds with potential anti-ALD activity included [41, 42]: (1) 0 PAINS alerts, (2) 0 violations of the Lipinski Rule of Five, (3) HIA < 0.7, (4) Fraction of oral bioavailability (f20%) < 0.7, (5) Plasma Protein Binding (PPB) ≤ 90%, (6) Ames test < 0.7, (7) Route of Administration (ROA) < 0.7, and (8) H-HT < 0.7. The widely accepted Lipinski Rule of Five [43–45], and human hepatotoxicity [44, 45] were specifically emphasized during the screening process.

#### Prediction of the potential drugable pockets for the target protein

To identify potential binding pockets on ALD-related targets, we first used the Proteins*Plus* platform (https://proteins.plus/pages/about) for protein structure analysis [46]. DoGSiteScorer was then applied to predict druggable binding sites [45]. Subsequently, SIENA was employed to assess whether oleanolic acid (PubChem CID: 10494), a key DRF compound, could fit into binding pockets across multiple AKT1 structures from the PDB [47]. This step aimed to evaluate the consistency and versatility of the binding site across different AKT1 conformations, offering insight into oleanolic acid’s interaction potential with AKT1 and supporting its therapeutic relevance for ALD.

#### Three-dimensional structural visualization of ligand-residue interactions

Visualization of 3D structures concerning Ligand-residue interactions between DRF compounds and selected targets was applied by Discovery Studio Visualizer 2024. For compounds anticipated to attach to locations within the druggable pocket, we assessed their total count of hydrogen bonds formed with the amino acid residues on the receptor protein.

#### Transcriptomic profiling and differential gene expression analysis

To explore ALD-related molecular changes, transcriptomic profiling was performed using the GEO dataset GSE167308 (7 alcoholic hepatitis patients, 5 healthy controls; platform: GPL20301). Raw count data were processed in R (v4.4.2) with DESeq2 (v1.38.0). Duplicate genes were averaged and mapped to unique gene symbols, and the count matrix was rounded to integers per DESeq2 requirements.

Low-abundance genes were filtered (≥ 1 count in ≥ 2 samples), and data were normalized using variance stabilizing transformation (VST). Group labels (‘CTRL’ vs. ‘ALD’) were inferred from filenames. Differential expression analysis was conducted using the Wald test (~ group), with Benjamini–Hochberg correction. DEGs were defined by |log_2_FC|> 1 and adjusted *p* < 0.05.

Results were visualized via ggplot2 (v3.5.1), including volcano plots with gradient log₂FC coloring and AKT1 annotation. Sample distribution was assessed by MDS, and gene labels were optimized using ggrepel (v0.9.5). Pipeline validation included duplicate-checking (anyDuplicated() = 0), checksum consistency, and reproducibility from intermediate files. Analyses were run in RStudio (v2024.12.0) using Quarto (v1.5.57) and tidyverse (v2.0.0) under macOS 10.15.7. A workflow summary and QC metrics are shown in Information S2.

#### Machine learning-based feature selection and classification

To identify key transcriptomic markers distinguishing ALD from controls, we applied Random Forest (RF), Support Vector Machine (SVM), and LASSO logistic regression using R (v4.4.2) in RStudio (v2024.12.0, macOS 10.15.7) with caret (v6.0–94), e1071 (v1.7–13), and glmnet (v4.1–8). RF was trained on gene expression and binary class labels (ALD = positive) using Leave-One-Out Cross-Validation (LOOCV). The mtry parameter was tuned for optimal AUC. Accuracy was validated by a binomial test (*p* < 0.05), and gene importance was assessed via mean decrease in Gini index. SVM with an RBF kernel was optimized via fivefold repeated cross-validation, using AUC as the criterion. The final model was evaluated with LOOCV, and gene importance was based on each feature's contribution to the decision boundary. LASSO was performed with α = 1 for L1 regularization. The optimal λ was chosen by minimizing LOOCV deviance. Genes with non-zero coefficients at λ_min were retained. AUC was calculated using LOOCV-predicted probabilities, and coefficient paths were plotted to assess feature stability. ROC curves were plotted for all models. The top 10 genes from each were selected by importance, and shared biomarkers across models were identified via Venn diagram analysis.

### Metabolite profiling and compound quantification

#### Untargeted metabolomics to analyze the active compounds of DRF

In addition to the TCMSP database, we identified active DRF components via untargeted metabolomics. DRF granules were sourced from Jiangyin Tianjiang Pharmaceutical Co., Ltd. (Wuxi, China), and the batch numbers of each herb were: Mentha haplocalyx (21092583), Pogostemon cablin (21100323), Portulaca oleracea (21093323), Taraxaci herba (21091793), Lophatheri herba (21092583), Lonicera japonica (21100813), Coicis semen (21101603), Myristica fragrans (21052583), and Phaseoli semen (21092733).

For sample prep, 50 mg of granules were extracted in 1 mL pre-chilled methanol–water (4:1, v/v) via homogenization and 20 min ultrasonication in ice. After 1 h at − 20 °C, samples were centrifuged (16,000 *g*, 20 min, 4 °C), and supernatants collected. Samples were analyzed on a Thermo Orbitrap Fusion^™^ Tribrid^™^ MS with a Waters UPLC® HSS T3 column (2.1 × 100 mm, 1.8 µm, 5 µL injection, 0.2 mL/min, 40 ℃). The gradient used solvent A (H₂O + 0.2% formic acid) and solvent B (acetonitrile): 0–8 min, 0% B; 8–45 min, 0–40% B; 45–50 min, 40–100% B; 50–60 min, 100% B; 60–60.1 min, 100–0% B; 60.1–70 min, 0% B.

Electrospray ionization (ESI) was used in both positive and negative modes. QE Plus MS parameters included: spray voltage 3.8 kV (+)/3.2 kV (−), capillary temp 320 °C, sheath gas 30, auxiliary gas 5, probe heat 350 °C, S-Lens RF 50. MS scan range was 80–1200 m/z, resolution 70,000, AGC 3e6, max IT 100 ms. Top 10 precursor ions were selected for MS/MS (resolution 17,500, AGC 1e5, max IT 50 ms, HCD, isolation window 2 m/z, stepped NCEs 20/30/40).

Data were processed using Thermo Xcalibur 2.2 and Compound Discoverer 3.3 for peak alignment, RT correction, and compound ID. Identification relied on accurate mass (10 ppm tolerance), MS1 (0.01 Da) and MS2 (0.02 Da) spectral matching, and fragment similarity > 0.70, with structure assignment based on ChemSpider, MassList, mzCloud, and mzVault databases.

#### HPLC-based quantification of oleanolic acid in DRF

To quantify oleanolic acid in DRF extract, high-performance liquid chromatography (HPLC) was performed using an Agilent 1260 Infinity II system (Agilent Technologies, USA) with a quaternary pump, autosampler, and diode array detector. Five grams of DRF granules (half adult daily dose) were dissolved in 200 mL methanol, then extracted with 200 mL ethyl acetate (1:1, v/v). The upper layer was collected, ultrasonicated for 30 min at room temperature, and centrifuged (14,000 rpm, 15 min). The supernatant was concentrated at 50 °C via rotary evaporation to a viscous residue, which was redissolved in 10 mL methanol, vortexed, and filtered through a 0.22 μm nylon filter. Chromatographic separation used a ZORBAX Eclipse XDB-C18 column (4.6 × 250 mm, 5 μm). The mobile phase: Solvent A (acetonitrile) and Solvent B (0.1% phosphoric acid in water), with a gradient: 0–30 min, 60–100% A; 30–35 min, hold at 100% A; 35–43 min, return to 60% A. Flow rate: 1.0 mL/min; column temp: 30 °C; detection wavelength: 200 nm; injection volume: 10 μL. A standard solution of oleanolic acid (purity > 98%, MedChemExpress) was prepared at 1.0 mg/mL in methanol. Calibration solutions (3.125–50 μg/mL) yielded a standard curve with R^2^ > 0.999.

### In vivo and in vitro experiments

#### Materials and chemicals

Oleanolic acid (purity > 98%) was obtained from MedChemExpress Bio-Technology Co., Ltd, and the positive control drug dexamethasone was sourced from Yuanye Bio-Technology Co., Ltd, both located in Shanghai, China. Absolute ethanol was purchased from Damao Chemical Reagent in Tianjin, China. The DMEM/F-12 high-glucose medium was supplemented with 10% fetal bovine serum (FBS), 1% penicillin–streptomycin, and insulin-transferrin-selenium (ITS), while the DMEM/F-12 basal medium was sourced from Pricella (Wuhan, China), FBS was acquired from Innochem Technology (Beijing, China), penicillin–streptomycin from Procell Life Science & Technology Co., Ltd. (Wuhan, China), and ITS was obtained from Beyotime Biotechnology (Shanghai, China). Dimethyl sulfoxide (DMSO) was purchased from Phthonbio in Nanjing, China, and the Cell Counting Kit-8 (CCK-8) was obtained from Biosharp Life Sciences (Hefei, China). Oil Red O was purchased from Aladdin Biochemical (Shanghai, China), isopropyl alcohol (IPA) from Baishi Chemical (Tianjin, China), and 4% paraformaldehyde fixative (PFA) from Biosharp Life Sciences (Hefei, China). The primary antibody against AKT1 was purchased from Cell Signaling Technology, Inc. (Danvers, MA, USA). All reagents were commercially available and of analytical grade. Specific batch numbers, catalog details, and additional supplier information are provided in the Information S3.

#### Cell culture and viability assay

AML12 cells, kindly gifted by Associate Professor Yanting You of Southern Medical University, were cultured in DMEM/F-12 high glucose medium supplemented with 10% FBS, 1% penicillin–streptomycin, and ITS at 37 °C under 5% CO_2_ conditions. For the viability assay, AML12 cells were seeded in 96-well plates at a density of 5,000 cells/well and allowed to adhere overnight. The cells were then treated with oleanolic acid (0–20 μM) for 24 h to assess its cytotoxicity using the CCK-8 assay.

#### Establishment of ALD cell model and experimental grouping

AML12 cells were seeded into 96-well plates at a density of 5,000 cells per well and incubated overnight to allow cell attachment. The ALD model was established by treating the cells with 2.5% ethanol-containing medium. The experiment was divided into six groups: (1) Control group (no ethanol, no treatment), (2) Model group (2.5% ethanol), (3) Low-dose treatment group (2.5 μM oleanolic acid), (4) Medium-dose group (5 μM oleanolic acid), (5) High-dose group (10 μM oleanolic acid), and (6) Positive control group (10 μM dexamethasone). Oleanolic acid and dexamethasone were dissolved in DMSO, and the final DMSO concentration in each well was maintained below 0.1%, which is considered non-toxic to cells; therefore, no vehicle control was included. Following model induction and treatment, cell viability was assessed after 6 h using the appropriate viability assay.

#### Zebrafish experiments

AB strain zebrafish (*Danio rerio*) were provided by the Zebrafish Modeling and Drug Screening Institute at Southern Medical University (Guangzhou, China). All experiments followed the Institutional Animal Care and Use Committee’s guidelines and adhered to the Animal Research: Reporting of In Vivo Experiments guidelines [48] (Supplementary Information S1). Zebrafish were cultured at 28 °C in water containing 200 mg/L sea salt, with a conductivity of 450–550 μS/cm, pH 6.5–8.5, and hardness of 50–100 mg/L CaCO_3_, under a 14 h light/10 h dark cycle. Embryos were obtained through natural pair-mating of adult zebrafish. To reduce selection bias, larvae were randomly assigned to treatment and control groups using a random number generator. The allocation of treatments to each well was also randomized. Personnel involved in morphological and physiological assessments were blinded to group assignments. Potential confounders such as tank location and environmental variation were minimized by rotating the plate positions during incubation.

#### Construction of the acute alcohol-induced liver injury zebrafish model

The acute alcohol-induced liver injury zebrafish model was established based on previously reported methods [49, 50]. Briefly, 3 days post-fertilization (dpf) zebrafish larvae were exposed to 2% ethanol for 36 h to induce liver injury. Dexamethasone was used as a positive control. A total of six experimental groups were set up: (1) control group, (2) model group (2% ethanol), (3–5) ALD model groups treated with low, medium, and high concentrations of oleanolic acid (2.5, 5, and 10 μM, respectively), and (6) ALD model group treated with dexamethasone (100 μM). Oleanolic acid was dissolved in DMSO to prepare a 10 mM stock solution, and appropriate volumes were added to fish water to achieve the desired concentrations. For example, 3 μL of 10 mM OA was added into 3 mL of fish water to obtain a final concentration of 10 μM. This ensured that the final concentration of DMSO did not exceed 0.1%, a level considered non-toxic to zebrafish larvae.

Morphological changes, heart rate, and malformation rates were observed and recorded under a stereomicroscope (Olympus MVX10 Microscope). Each group contained three technical replicates (n = 3 wells), with 20 larvae per replicate, placed in individual wells of 6-well plates. The total number of larvae per group was 60 (n = 60), and the total number across the experiment was 360.

Larvae were selected from the same spawning batch, and those with pre-existing malformations were excluded before treatment. During the experiment, all larvae within each well were included in the analysis of morphological changes and malformation rates. Heart rate was assessed by randomly selecting three larvae per well, resulting in nine measurements per group. Morphological assessments and heart rate measurements were performed under a stereomicroscope (Olympus MVX10). Representative images of zebrafish were captured using cellSens software (version 2.2), which was also used to determine body length parameters. All outcome assessments were conducted by trained personnel blinded to the treatment allocation. Primary endpoints included morphological abnormalities, heart rate, and malformation rates. Heart rate was measured by manually counting beats per minute under the microscope.

#### Oil red O staining

To assess Lipid accumulation in 4.5 days dpf zebrafish larvae, larvae were first anesthetized in 0.02% Tricaine solution. Fixation was performed in 4% paraformaldehyde (PFA) at 4 ℃ for 24 h, followed by three washes with 1 × phosphate-buffered saline (PBS) for 5 min each. Larvae were then dehydrated through a graded series of propylene glycol (20%, 40%, 80%, and 100%), 5 min per step. Subsequently, the larvae were stained in Oil Red O working solution (0.5% Oil Red O in isopropanol) at 60 ℃ for 1 h in the dark. After staining, excess dye was removed by decolorization using a gradient of propylene glycol (100%, 80%, 40%, and 20%) from high to low, 5 min per step. Then rinse three times with PBS, each time for 5 min.

#### Cellular thermal shift assay

The interaction between oleanolic acid and AKT1 was evaluated using Cellular Thermal Shift Assay (CETSA). AML12 cells were harvested and lysed using RIPA buffer supplemented with protease and phosphatase inhibitors. The lysates were divided into two equal aliquots: one incubated with oleanolic acid (at a final concentration equivalent to one-tenth of the total protein concentration), and the other treated with DMSO as vehicle control. After 2 h of incubation at 4 °C, the mixtures were aliquoted into PCR tubes and subjected to a temperature gradient (40–70 °C, at 8 °C intervals) for 3 min using a thermal cycler. Samples were then cooled to room temperature and centrifuged at 14,000 × *g* for 20 min at 4 °C to separate soluble fractions. Supernatants were collected and analyzed by western blotting. Proteins were separated via SDS-PAGE and transferred onto PVDF membranes. After blocking, membranes were incubated with anti-AKT1 primary antibody overnight at 4 °C, followed by HRP-conjugated secondary antibody. Bands were visualized using chemiluminescence and quantified using ImageJ. The thermal stability of AKT1 was compared between oleanolic acid-treated and control groups to assess compound-target engagement.

#### Statistical analysis

For comparisons between two groups, unpaired two-tailed Student’s t-tests were used. Before performing the t-test, the assumption of normality was checked using the Shapiro–Wilk test, and the homogeneity of variance was verified using Levene’s test. Data are presented as mean ± standard deviation, and *p* < 0.05 was considered statistically significant.

## Results

### Systems-level network dissection of DRF-ALD crosstalk via integrative target mapping and functional enrichment analysis

**Fig. 1:**
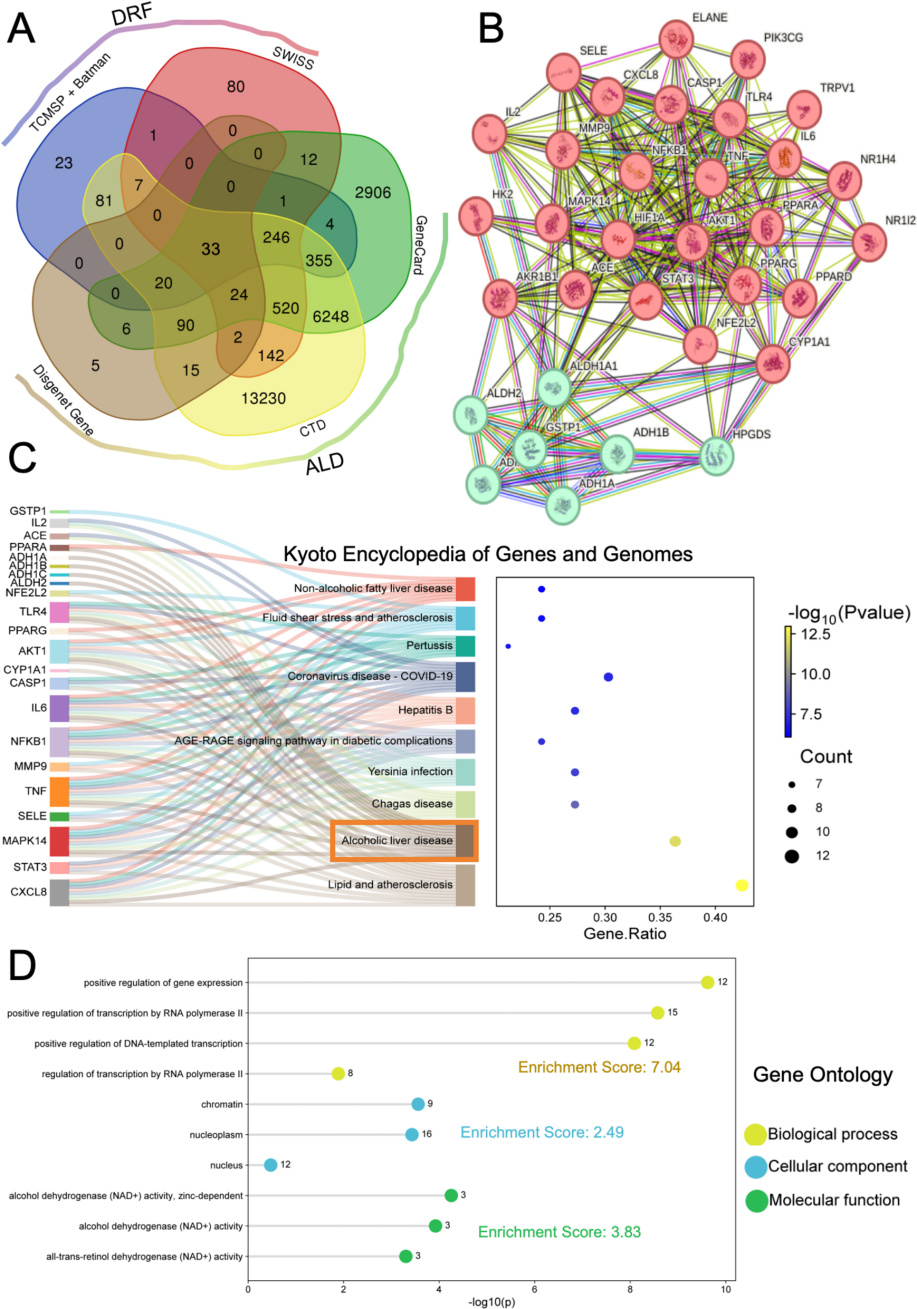
Network Pharmacology Analysis of Dampness-Heat Regulating Formula in Treating Alcoholic Liver Disease. **A** Venn diagram showing the intersecting targets between Dampness-Heat Regulating Formula and Alcoholic Liver Disease. **B** Protein–Protein Interaction network of the identified targets. **C** Kyoto Encyclopedia of Genes and Genomes pathway enrichment analysis for the identified targets. **D** Gene Ontology enrichment analysis categorizing biological processes, molecular functions, and cellular components associated with the identified targets

To decipher the therapeutic mechanisms of the DRF in ALD, a systems-level strategy integrating multi-database compound-target mapping and functional relevance analysis was employed. Initially, 690 candidate compounds (Table S1) were retrieved from the TCMSP database. Specifically, active compounds from *Taraxacum mongolicum* and *Lophatherum gracile* were additionally obtained through BATMAN-TCM, while those from the remaining DRF components were sourced from TCMSP. Predicted targets were then cross-validated using SwissTargetPrediction, and intersecting targets across platforms were retained as high-confidence DRF-associated targets. Concurrently, ALD-related targets were curated from GeneCards, CTD, and DisGeNET databases. Only targets present in all three databases were selected to ensure disease relevance and data robustness. The intersection of these disease targets with the DRF compound targets yielded 33 overlapping core targets (Fig. 1A), representing putative mediators of DRF’s pharmacological activity against ALD.

Protein–protein interaction (PPI) analysis was conducted via the STRING database, generating a high-confidence network of 33 nodes and 248 edges (average node degree = 15, clustering coefficient = 0.726, PPI enrichment *p* < 1.0e-16), substantially exceeding the expected 78 edges. This enrichment suggests a non-random, functionally interconnected protein network potentially underpinning the synergistic action of DRF (Fig. 1B). Subsequent Markov Clustering Algorithm (MCL) identified two dominant clusters: Cluster 1 (26 targets) involved in Lipid localization regulation, and Cluster 2 (7 targets) related to ethanol oxidation—highlighting core pathological features of ALD addressed by DRF.

KEGG pathway enrichment (Fig. 1C and Table S3) further supported these findings, with top-ranking pathways including ‘Lipid and atherosclerosis’ and ‘Alcoholic liver disease’. Key targets such as TNF, IL6, and NFKB1 were enriched, implying that DRF may exert therapeutic effects through modulation of lipid homeostasis and inflammatory cascades—both hallmarks of ALD pathogenesis. Bubble plot visualizations encoded pathway significance (*p*-values) and target abundance, providing a visual cue of pathway prioritization. To complement pathway-level insights, Gene Ontology (GO) enrichment analyses were conducted across Biological Process (BP), Cellular Component (CC), and Molecular Function (MF) dimensions (Fig. 1D). BP enrichment highlighted ‘positive regulation of gene expression’ (cluster score = 7.04), indicating transcriptional reprogramming as a possible mechanism. The top CC term, ‘nucleoplasm’ (score = 2.49), suggests nuclear-localized activity of several targets, while MF analysis identified ‘alcohol dehydrogenase (NAD^+^) activity, zinc-dependent’ (score = 3.83), directly linking to ethanol metabolism and detoxification.

### High-throughput molecular docking screening of DRF compounds targeting core proteins in ALD pathways

Based on the results of the KEGG clustering analysis, we performed a node degree ranking of 12 proteins involved in the ALD pathway using the STRING database (Table S3). From this ranking, we selected the top five proteins with the highest node degree values as our key research targets: RAC-α serine/threonine-protein kinase 1 (AKT1), C-X-C motif chemokine Ligand 8 (CXCL8), IL6, Toll-like receptor 4 (TLR4), and TNF. These proteins demonstrated the highest connectivity within the network, indicating their potential central roles in the pathway. Subsequently, each of these five target proteins was subjected to molecular docking with a Library of 690 compounds from the DRF to explore their potential interactions and therapeutic relevance.

**Fig. 2:**
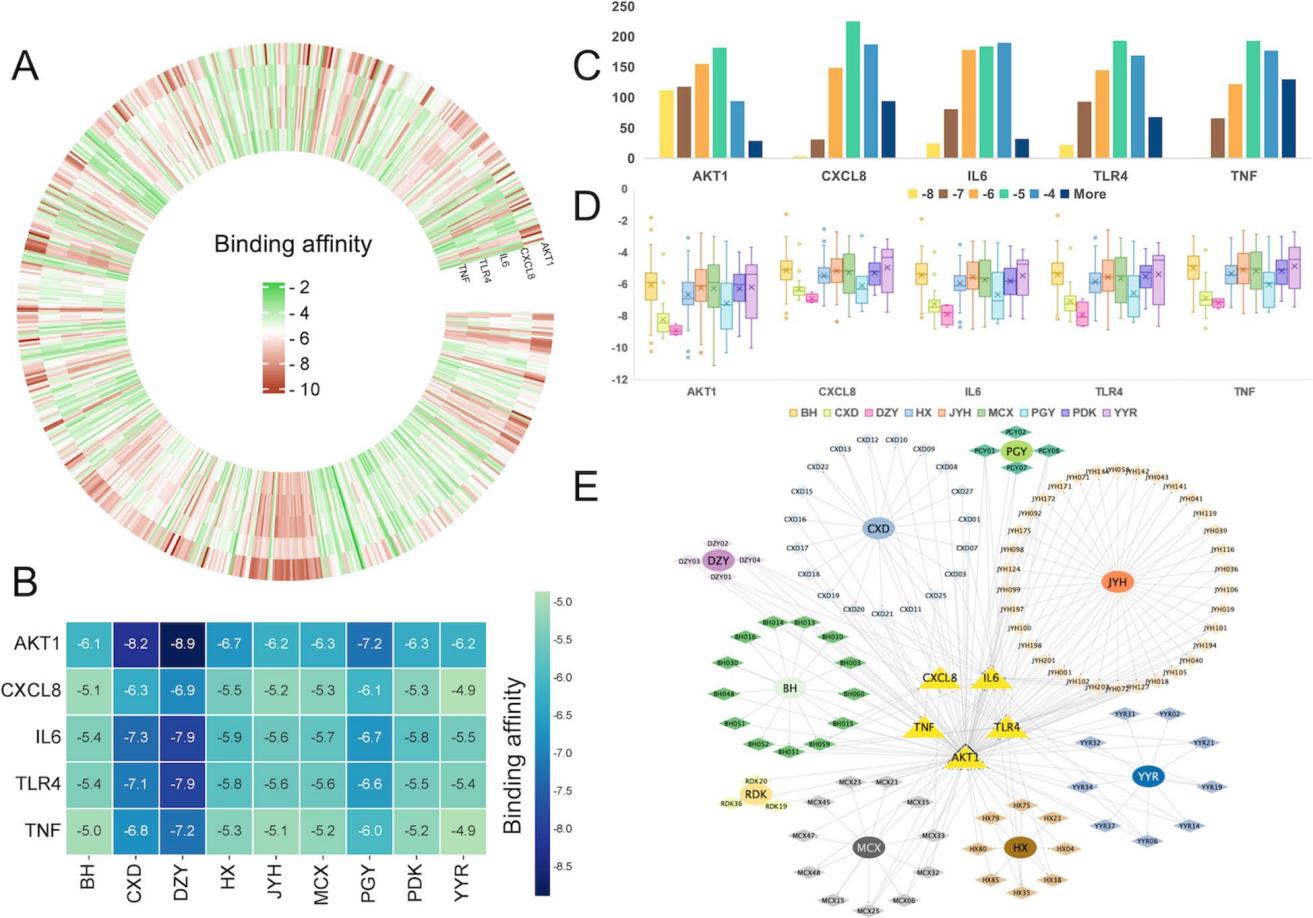
High-throughput All-Atom Molecular Docking Analysis of Dampness-Heat Regulating Formula. **A** Overall docking results. **B** Average binding affinities of active compounds grouped by their respective herbs in the Dampness-Heat Regulating Formula. **C** Bar chart showing the docking binding energies of Dampness-Heat Regulating Formula compounds with target proteins. **D** Box plot illustrating the docking binding energies of individual herbs in Dampness-Heat Regulating Formula. **E** Network plot of compounds with binding affinities lower than − 8 kcal/mol, showing their interactions with various target proteins

A total of 690 components from the nine herbs in DRF were docked against the five core protein targets, resulting in 3450 molecular docking outcomes (Table 1, Fig. 2A and Table S5). It is widely accepted that lower binding energy values indicate a more stable conformation between the active constituents and the target receptors, thereby indicating a higher likelihood of binding. The binding scores ranged from −1.6 to − 11.1 kcal/mol, with an average affinity of − 5.6 kcal/mol. Notably, 1,303 of the docking results (37.77%) exhibited binding energies ≤ − 6.0 kcal/mol. Among the molecular docking results, the interaction between AKT1 and DRF was found to be the most efficacious, exhibiting a range of binding energies from − 1.8 to − 11.1 kcal/mol, with an average binding energy of − 6.36 kcal/mol. Following closely were the interactions with IL6 and TLR4, which displayed average binding energies of − 5.7 and − 5.6 kcal/mol, respectively, and binding energy ranges of − 1.9 to − 8.8 kcal/mol and − 1.7 to − 8.9 kcal/mol, respectively. In contrast, the least effective docking was observed for TNF, with an average binding energy of − 5.2 kcal/mol and a binding energy range spanning from − 1.8 to 8.8 kcal/mol.

**Table 1:** Details of docking results between 690 natural compounds from Dampness-Heat Regulating Formula and five targets for alcoholic liver disease (kcal/mol)

Target name	Total binding score	Min	25% percentile	Med	75% percentile	Max	Ave	SD	SEM	95% confidence interval of mean		Top-1 compound
										Lower	Upper	
AKT1	− 4382.3*	− 11.1	− 7.3	− 6.2	− 5.3	− 1.8	− 6.4	1.5	0.1	− 6.3	− 6.4	MCX25
CXCL8	− 3636.7	− 8.4	− 6.1	− 5.2	− 4.5	− 1.6	− 5.3	1.1	0.1	− 5.3	− 5.3	JYH072
IL6	− 3926.1	− 8.8	− 6.5	− 5.7	− 4.8	− 1.9	− 5.7	1.2	0.1	− 5.7	− 5.7	JYH072, CXD22
TLR4	− 3882.7	− 8.9	− 6.5	− 5.6	− 4.7	− 1.7	− 5.6	1.3	0.1	− 5.6	− 5.6	JYH072
TNF	− 3581.9^**#**^	− 8.8	− 6.1	− 5.2	− 4.3	− 1.8	− 5.2	1.2	0.1	− 5.2	− 5.2	CXD25

The target name in the first column represents the targets of hyperuricemia and each target is given a new ID name in the first column. The bold value refers to the target with best total binding score; * the maximum value; ^**#**^ the minimum value; *Min*, minimum; *Med*, median;*Max*, maximum; *Ave,* average; *SD*, standard deviation; *SEM*, standard error of means

As shown in Fig. 2B, the average binding affinities of the DRF ingredients to various core targets were revealed, highlighting the differential interactions between the ingredients and their respective targets. The data indicate that DZY (*Lophatheri herba*) demonstrates the strongest binding affinity across all targets, with binding energies ranging from − 5.8 kcal/mol (for TNF) to − 8.9 kcal/mol (for AKT1), suggesting its significant potential as a therapeutic agent. CXD (*Phaseoli semen*) shows good binding, with values ranging from − 5.3 kcal/mol (for TNF) to − 7.2 kcal/mol (for AKT1), slightly weaker than DZY, but still indicating strong therapeutic potential. PGY (*Taraxaci herba*) also displays favorable binding, with values between − 5.2 kcal/mol (for TNF) and − 7.2 kcal/mol (for AKT1), supporting its role in the treatment of ALD. On the other hand, HX (*Pogostemon cablin*) and YYR (*Coicis semen*) exhibit weaker binding affinities, with values ranging from − 5.1 kcal/mol to − 5.4 kcal/mol, suggesting that while they show some potential, they are less effective compared to the other compounds. These findings demonstrate that DZY, with its superior binding affinity, holds the most promise as a potential therapeutic agent for ALD treatment.

Binding patterns were analyzed using histograms (Fig. 2C) to provide an overview of how key targets interact with DRF compounds [42]. AKT1 exhibits the highest number of results with binding energies below − 8 kcal/mol, totaling 112, while it has the fewest results with higher binding energies, amounting to only 29. This indicates that DRF demonstrates a superior docking affinity for the protein target AKT1 compared to the other targets, resulting in the most stable binding conformation. In contrast, IL6 and TLR4 perform poorly relative to the other molecular targets, showing a greater number of results with high binding energies.

The binding stability of these compounds is depicted in a boxplot, with interquartile ranges indicating data concentration and identifying any outliers (Fig. 2D). A smaller interquartile range signifies more concentrated data and stronger binding affinity for a higher number of compounds, while wider ranges suggest the presence of weaker binding compounds. Despite the good performance of AKT1 relative to other protein targets in terms of average binding energy, it was shown in Fig. 2D to have a large outlier range for binding to DRF, suggesting the presence of weak binding compounds and therefore a relative lack of specific targeting by DRF’s components. From the perspective of DRF, the average binding energy levels of each group of traditional Chinese medicine molecules with protein targets are concentrated around − 5 to − 7 kcal/mol. The top three herbs with the best binding effect were DXY (− 7.76 kcal/mol), CXD (− 7.16 kcal/mol) and PGY (− 6.51 kcal/mol). Notably, the docking results for the compounds of CXD and DZY with each protein target were significantly superior to those of others, both in terms of average binding energy (− 7.16 kcal/mol and − 7.76 kcal/mol) and the concentration of binding energy. This indicates that these two TCMs bind to the ALD-related protein target more stably and are more targeted. In contrast, the compounds of YYR exhibited lower mean values and larger outlier ranges for each target docking display compared to other groups, indicating a slightly inferior binding ability to protein targets.

The herb-compound-target network diagram (Fig. 2E) revealed that DRF contains a complex multi-target interaction profile, highlighting the synergistic effects of its components in modulating key biological pathways associated with ALD. Overall, AKT1 emerged as the core target with the strongest binding affinity among the five key proteins, exhibiting the highest number of stable interactions with DRF compounds, particularly at binding energies below − 8 kcal/mol. This suggests that AKT1 may play a central role in DRF’s mechanism of action against ALD, potentially serving as a primary target for modulating disease pathways. Notably, the herbs DZY and CXD demonstrated consistently strong binding affinities across multiple protein targets, including AKT1, IL6, and TLR4. The robust interactions of DZY and CXD compounds with these targets, especially AKT1, highlight their potential contributions to the therapeutic efficacy of DRF. These findings suggest that DZY and CXD may be key components within DRF, with their active compounds exhibiting both stable and specific binding to ALD-related protein targets, thereby enhancing DRF’s capacity to regulate critical pathways associated with ALD.

### Cluster analyses of 690 compounds in DRF

Given that the compound structure significantly influences interactions with the target, we analyzed the structural properties of 690 compounds in the DRF related to ALD and their target, AKT1. Our focus was on elucidating the relationship between structural characteristics and binding affinity.

**Fig. 3:**
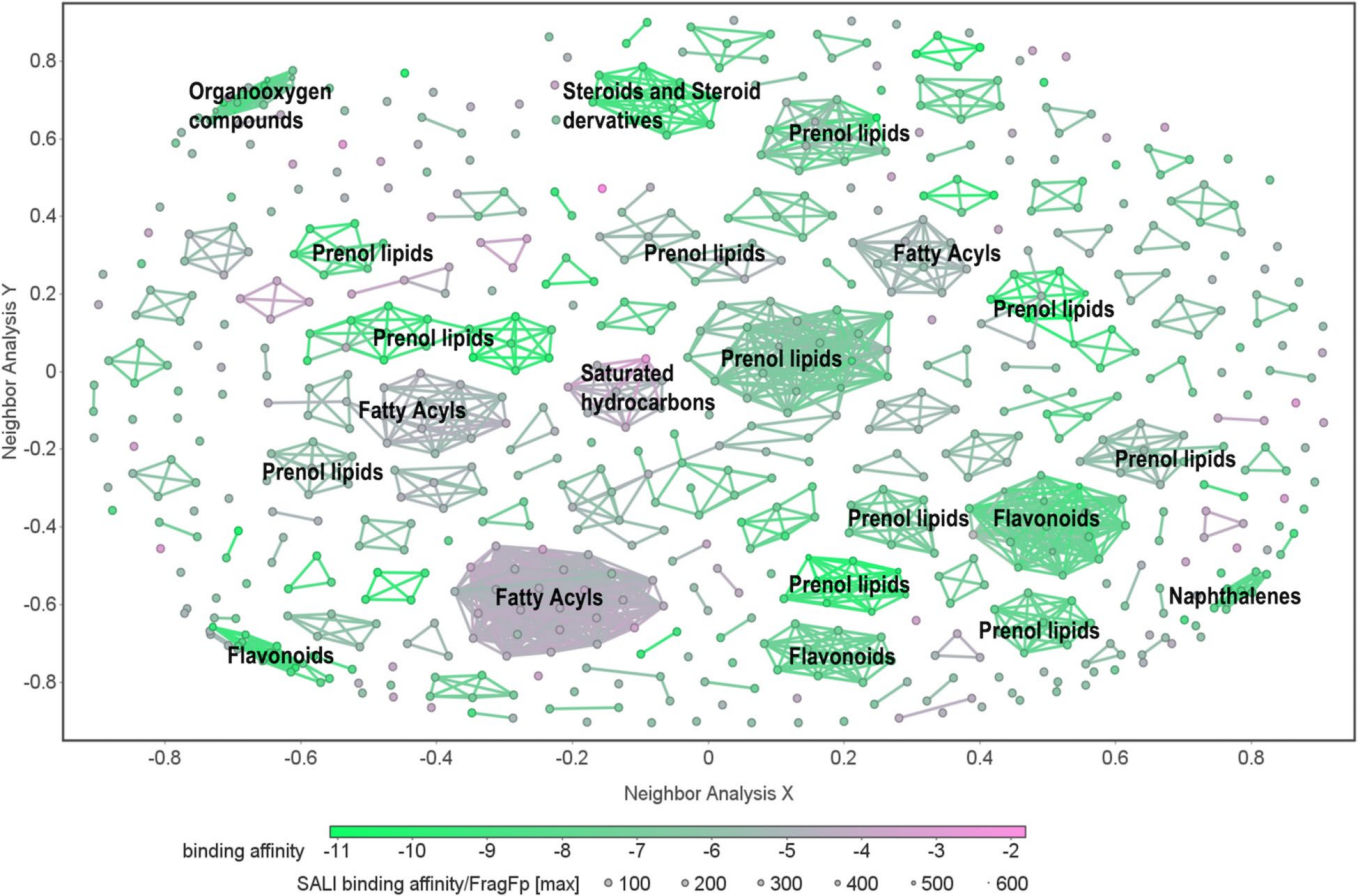
The cluster network of Dampness-Heat Regulating Formula based on structural similarity. Each node represents a drug-resistant compound, and edges connect structurally similar compounds. Node color corresponds to AKT1 binding affinity, while node size integrates both binding affinity and structual similartiy: larger nodes indicate lower affinity and highter similarity to neighboring compounds

As illustrated in the clustering network (Fig. 3), the colors represent the binding scores of the 690 compounds of the DRF to AKT1, with binding energy decreasing as the color shifts toward green. Structurally similar compounds are grouped, and the consistent colors within these clusters indicate a strong relationship between the ligand’s structure and its interaction capabilities. Our focus is on the clusters of compounds containing more than seven nodes.

The cluster network of 690 compounds of DRF (Fig. 3) reveals the presence of 22 clusters, each containing more than seven compounds. Based on the clustering results, we identified that these compounds are primarily prenol Lipids, fatty acyls, flavonoids, and Organooxygen compounds, with Prenol Lipids being the most abundant. The cluster with the largest node comprised 46 compounds, of which over 70% were prenol lipids. Notably, among the four clusters exhibiting low binding energy and high affinity for AKT1, two were composed of prenol lipids, while the other two clusters consisted of steroids and steroid derivatives, and Naphthalene derivatives, respectively.

In the cluster analysis conducted on a group of compounds derived from each herb of DRF (Fig. S1), it was observed that JHY formed the most clusters. The groups with more clusters may indicate a greater diversity in molecular categories, suggesting that the compounds within JHY could exert a broader spectrum of biological effects. The two clusters of compounds with the lowest binding energies in the cluster analysis of JHY (Fig. S1A) were identified as organooxygen compounds and flavonoids. The molecules of CXD formed only two clusters (Fig. S1D), with the average binding energies of both clusters being less than −8 kcal/mol; the predominant compounds in these clusters were prenol lipids. Similarly, HX also formed two clusters (Fig. S1C), each containing more than seven nodes, with the primary compounds in both clusters being prenol lipids and flavonoids.

### Chemoinformatic clustering reveals structure-affinity relationships of DRF compounds against AKT1

Six compounds were identified that met all the ADMET screening criteria: RDK59 (Saucernetindiol), JYH039 (Hederagenol), JYH163 (2-(2,4-Dimethoxyphenyl)−3-hydroxy-7-methoxy-chromone), JYH172 (Akebiasaponin D_qt), BH048 (Oleanolic acid), and BH060 (Ursolic acid). A detailed comparison of the ADMET properties of these six compounds is shown in Table 2.

**Table 2:** Detailed comparison of the ADMET properties of the potential six active compounds

Compound code	Molecule name	PAINS	Lipinski	HIA	f20%	PPB	Ames	ROA	H-HT
RDK59	Saucernetindiol	Pass	0 alert	0.00	0.09	89.95	0.55	0.48	0.50
JYH039	Hederagenol	Pass	0 alert	0.06	0.08	75.78	0.05	0.13	0.43
JYH163	2-(2,4-Dimethoxyphenyl)−3-hydroxy-7-methoxy-chromone	Pass	0 alert	0.52	0.04	87.50	0.68	0.35	0.45
JYH172	Akebiasaponin D_qt	Pass	0 alert	0.00	0.69	78.79	0.12	0.15	0.53
BH048	Oleanolic acid	Pass	0 alert	0.01	0.01	87.10	0.04	0.21	0.41
BH060	Ursolic acid	Pass	0 alert	0.01	0.00	86.61	0.03	0.12	0.41

*H-HT* Human Hepatotoxicity, *PAINS* Pan Assay Interference Compounds, *HIA* Human Intestinal Absorption, *f20%* 20% Fraction unbound in plasma, *PPB* Plasma Protein Binding, *Ames* Ames test for mutagenicity, *ROA* Route of Administration. Values of H-HT, AMES toxicity, and carcinogenicity represent probabilities. When the results of the above three indicators are less than or equal to 0.5, they are considered as pass. Compounds were considered to meet the screening criteria if they had 0 PAINS alerts, 0 Lipinski violations, and values of HIA, f20%, Ames, ROA, and H-HT < 0.7, with PPB ≤ 90%. All data were retrieved from ADMETlab 2.0 (https://admetmesh.scbdd.com/)

The ADMET screening results reveal that all six compounds satisfy the predefined thresholds, showing no PAINS alerts or violations of the Lipinski Rule of Five. The HIA values of the compounds vary, with JYH163 showing the highest absorption potential (0.5182) and JYH172 having the lowest (0.00076). Similarly, the f20% values indicate acceptable oral bioavailability for all compounds, with values ranging from 0.0037 (BH060) to 0.6926 (JYH172). Regarding Plasma Protein Binding (PPB), all compounds exhibit values below 90%, suggesting favorable binding properties. The compound with the lowest PPB is JYH039 (75.78%), while RDK59 displays the highest PPB (89.95%). For Ames test and ROA, all compounds demonstrate low toxicity risks, with BH060 achieving the lowest Ames score (0.0310). Finally, Hepatotoxicity (H-HT) values are all ≤ 0.7, indicating low risks of liver toxicity. BH048 (Oleanolic acid) shows the lowest hepatotoxicity value (0.4139), further supporting its potential safety as a therapeutic agent for ALD.

**Fig. 4:**
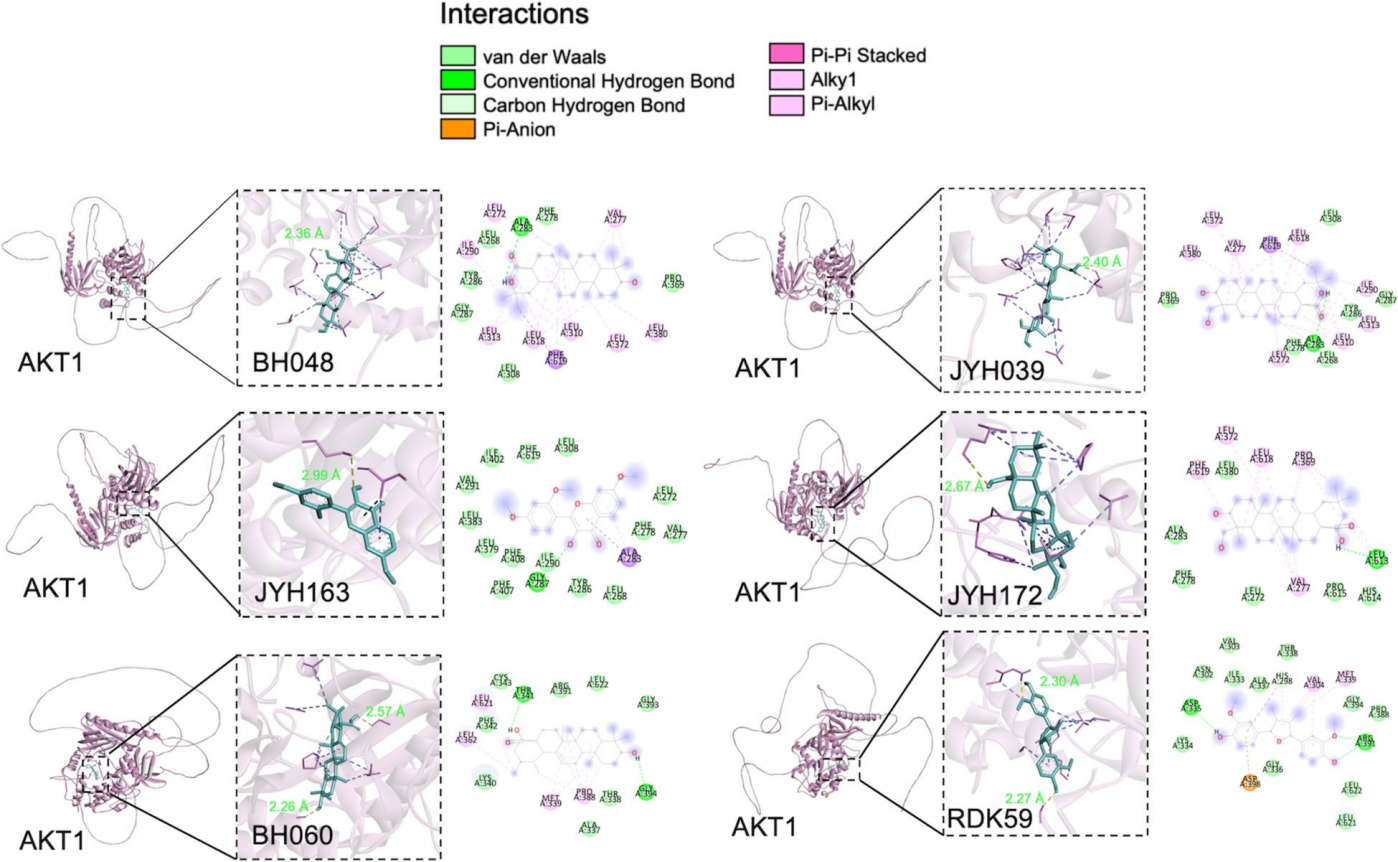
The binding modes of six ligands from Dampness-Heat Regulating Formula with AKT1. 3D and 2D Docking Visualizations shows the AKT1 protein (Pink) with ligand binding sites (blue). The lower section details ligand interactions (green) with AKT1 amino acids via H-bonds (green lines) or other interactions, with bond distances in Å

All six compounds exhibited strong binding interactions with AKT1 (Table 3 and Fig. 4), forming hydrogen bonds with key residues. RDK59 (Saucernetindiol) formed two hydrogen bonds with residues ARG391 and ASP335, with bond lengths of 2.30 Å and 2.27 Å, respectively. JYH039 (Hederagenol) formed one hydrogen bond with residue ALA283, with a bond length of 2.40 Å. JYH163 (2-(2,4-dimethoxyphenyl)−3-hydroxy-7-methoxy-chromone) formed one hydrogen bond with residue GLY287, with a bond length of 2.99 Å. JYH172 (Akebiasaponin D_qt) formed one hydrogen bond with residue LEU613, with a bond length of 2.67 Å. BH048 (oleanolic acid) formed one hydrogen bond with residue ALA283, with a bond length of 2.36 Å, and BH060 (Ursolic acid) formed two hydrogen bonds with residues THR341 and GLY394, with bond lengths of 2.57 Å and 2.26 Å, respectively. Binding affinity values ranged from −7.1 kcal/mol (JYH163) to − 10.2 kcal/mol (BH048), indicating strong binding potential for all six compounds. BH048 (Oleanolic acid) exhibited the highest binding affinity (− 10.2 kcal/mol), followed by BH060 (Ursolic acid) (− 8.9 kcal/mol). These findings highlight the potential of the selected compounds to interact effectively with AKT1, supporting their candidacy for further exploration as therapeutic agents against ALD.

**Table 3:** Detailed comparison of the ADMET properties of the potential six active compounds

Compound code	Molecule name	Binding affinity (kcal/mol)	Number of H-bonds	Binding residue
RDK59	Saucernetindiol	− 7.7	2	ARG391, ASP335
JYH039	Hederagenol	− 9.6	1	ALA283
JYH163	2-(2,4-Dimethoxyphenyl)−3-hydroxy-7-methoxy-chromone	− 7.1	1	GLY287
JYH172	Akebiasaponin D_qt	− 8.2	1	LEU613
BH048	Oleanolic acid	− 10.2	1	ALA283
BH060	Ursolic acid	− 8.9	2	THR341, GLY394

*H-bond* hydrogen bond

In conclusion, these six compounds were identified as promising candidates for further investigation due to their drug-like properties, low toxicity potential, and favorable ADMET profiles. Among them, JYH163 exhibited the best overall absorption and bioavailability characteristics, while BH048 and BH060 showed minimal hepatotoxicity, highlighting their potential for safe and effective treatment of alcoholic liver disease.

### Transcriptome-based and machine learning-supported validation of AKT1 as a core target in ALD

**Fig. 5:**
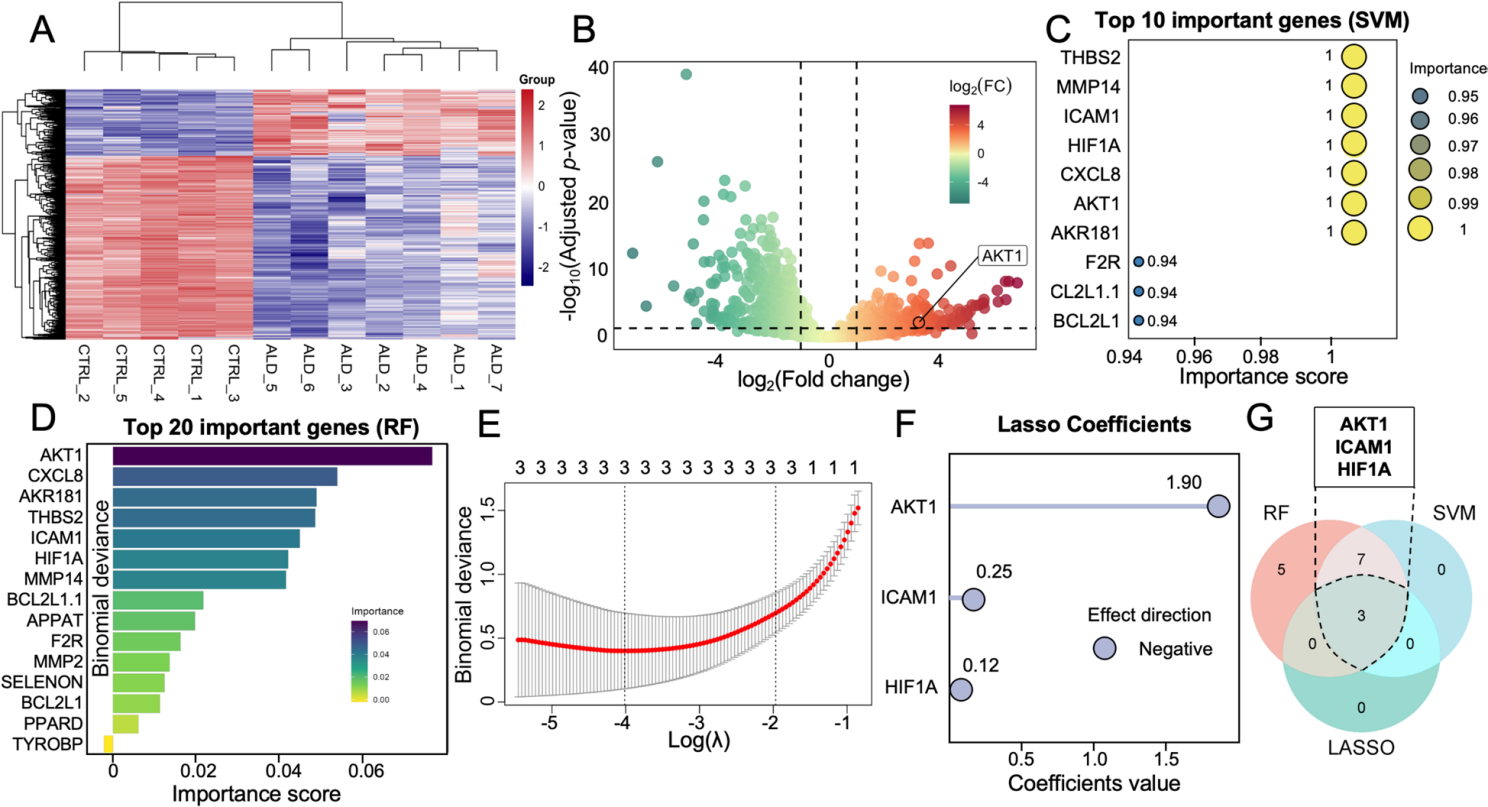
Transcriptomics-based machine learning identification of core genes related to DRF against ALD. **A** Heatmap of differentially expressed genes (DEGs) between healthy individuals and ALD patients, showing distinct clustering patterns. **B** Volcano plot highlighting significantly up- and down-regulated genes (adjusted *p* < 0.05, |log₂FC|> 1). **C** Top 10 important genes selected by support vector machine (SVM) model, ranked by classification contribution. **D** Top 15 important genes identified by random forest (RF), based on importance scores. **E** Feature selection by LASSO regression for gene dimensionality reduction. **F** LASSO coefficient plot indicating the direction and magnitude of gene contributions. **G** Venn diagram showing the intersection of top genes from three models

Transcriptomic analysis of hepatic tissue from ALD patients (GSE167308) identified 904 differentially expressed genes (DEGs) compared to healthy controls (|log_2_FC|> 1, adjusted *p* < 0.05), including 509 upregulated and 395 downregulated genes (Table S7). PCA revealed clear group separation (Fig. S2). Volcano plot visualization (Fig. 5B) highlighted AKT1 as significantly upregulated (log_2_FC = 3.23, adjusted *p* = 0.0007), consistent with its prioritization through network analysis and docking. Hierarchical clustering of the DEGs demonstrated distinct sample grouping and consistent within-group expression trends (Fig. 5A), supporting the transcriptional impact of ALD. These results provide strong transcriptomic evidence that AKT1 is a central, disease-relevant target likely involved in DRF-mediated therapeutic effects.

To further elucidate the most robust biomarkers from the upregulated DEGs, we employed three complementary machine learning approaches, each rigorously validated through LOOCV to address the challenges of small sample size. Random Forest analysis (Fig. 5D and Table S7) demonstrated perfect classification performance, achieving 100% accuracy (12/12 samples correctly classified) and an AUC of 1.0 (*p* = 0.003, binomial test), with AKT1 emerging as the top predictive gene (importance score = 0.0695), followed by CXCL8 (0.0488) and AKR1B1 (0.0443). The negative association of TYROBP (importance = − 0.002) suggested its potential role as an inhibitory factor in ALD progression. SVM modeling with a radial basis function kernel identified optimal parameters at C = 1 and σ = 0.1 (Table S9), yielding strong discriminative power (AUC = 0.96). The SVM algorithm (Fig. 5C and Table S8) highlighted CXCL8, AKR1B1, AKT1, ICAM1 and MMP14 as having maximum importance scores (1.000), with BCL2L1 and F2R also showing high predictive value (importance = 0.94). LASSO regression analysis (Fig. 5E and F) identified an optimal regularization parameter of λ = 0.017 (log(λ) = − 4.05), which selected three genes with non-zero coefficients: AKT1, ICAM1 and HIF1A (Table S9 and S10). The model showed excellent classification performance (AUC = 0.963) and remarkable stability across λ ranges, as evidenced by the regularization path plot.

The integrative analysis combining three machine learning approaches (Random Forest, SVM, and LASSO) consistently identified AKT1, ICAM1, and HIF1A as key biomarkers through Venn intersection, with AKT1 demonstrating superior performance across all evaluation metrics. This consistent top-ranking performance across three distinct algorithms, coupled with its optimal binding characteristics in our molecular docking studies (demonstrating the most favorable binding affinity and stability among the three candidates), strongly supports AKT1 as the most reliable and biologically relevant therapeutic target for ALD.

### Oleanolic acid alleviates ethanol-induced hepatotoxicity in vitro and in vivo

**Fig. 6:**
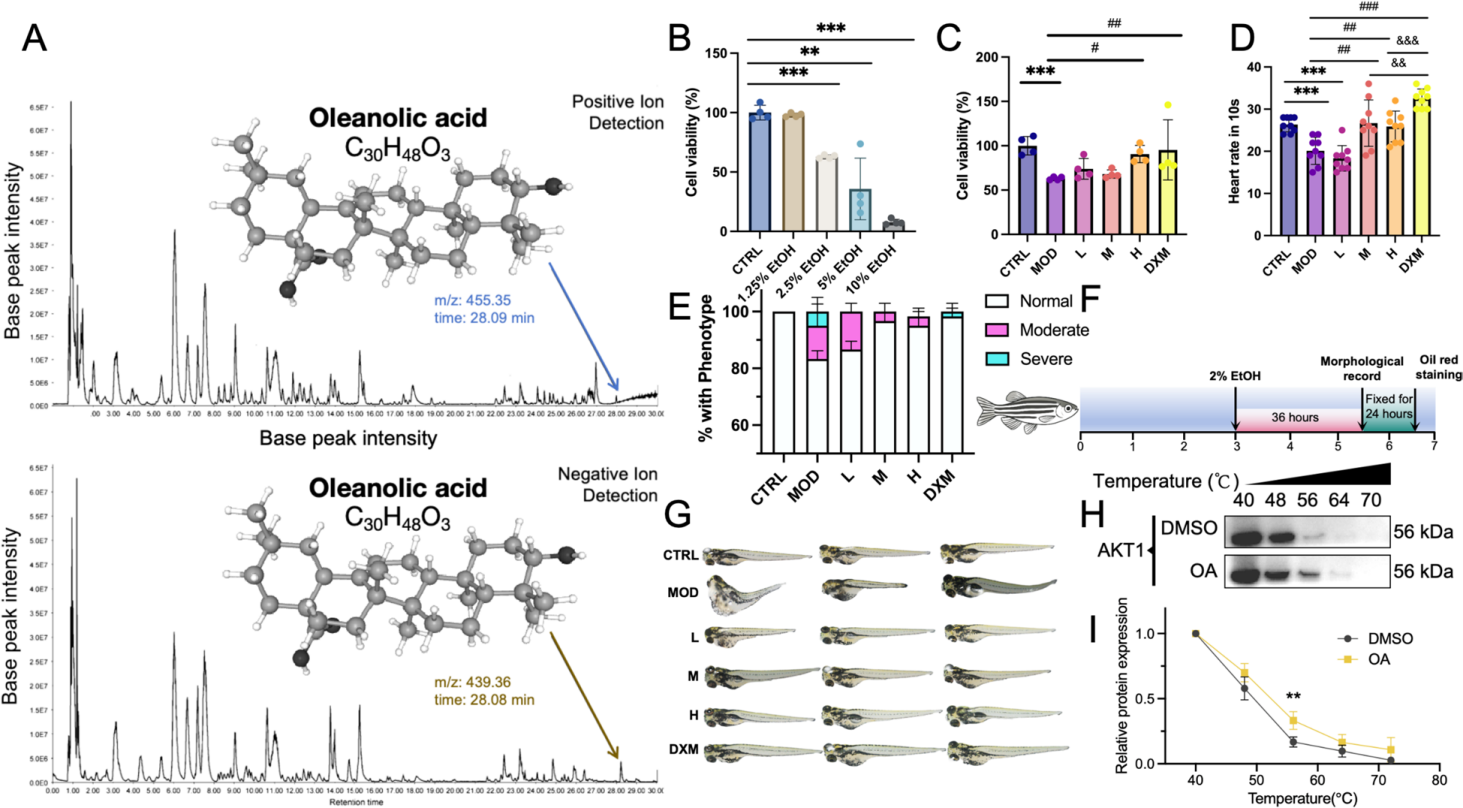
Oleanolic acid identified in DRF attenuates ethanol-induced hepatotoxicity in vitro and in vivo. **A** Untargeted metabolomics confirms the presence of oleanolic acid in DRF. **B** Viability of AML12 hepatocytes after 24 h ethanol exposure (1.25%–10%), identifying 2.5% as the optimal concentration for model induction. **C** Effects of oleanolic acid (2.5, 5, 10 μM) and dexamethasone (10 μM) on cell viability under 2.5% ethanol-induced injury. **D** Heart rate measurements of zebrafish larvae in each group (n = 9 per group, 3 larvae per replicate). **E** Phenotypic classification of zebrafish larvae following ethanol exposure, categorized as normal, moderate (uninflated swim bladder), or severe (swim bladder defect defect plus edema or body curvature). Data based on three replicates of 20 larvae (n=60). **F** Schematic overview of the zebrafish acute alcohol-induced liver injury model and treatment strategy. **G** Representative images of zebrafish morphology under different treatment conditions. **H** Western blot showing thermal stability of AKT1 after incubation with OA or DMSO across 40–70 °C. **I** Quantification of relative AKT1 levels, normalized to 40 °C. Data: mean ± SD, n = 4. Data are presented as mean ± SD. **p < 0.01, ***p < 0.001 vs. CTRL; #*p* < 0.05, ##*p* < 0.01, ###*p* < 0.001 vs. MOD; &&*p* < 0.01, &&&*p* < 0.001 between indicated groups. *L/M/H* low, medium, and high doses of oleanolic acid, *DXM* dexamethasone

To validate the presence of bioactive compounds in DRF, untargeted metabolomics analysis was performed using UPLC-Q-Exactive Plus (LC–MS/MS) in both positive and negative ion detection modes (Fig. 6A and Table S6). The base peak chromatograms provided a comprehensive chemical profile of the DRF samples. In the negative ion mode, a total of 1402 known compounds were detected, while 2019 known compounds were identified in the positive ion mode. Among the six potential compounds, oleanolic acid (C_30_H_48_O_3_, MW: 456.35979 Da) was detected in the negative ion mode with an m/z value of 455.35, and a retention time of 28.09 min. In the positive ion mode, the same compound was identified with an m/z value of 439.36, and a retention time of 28.08 min. Annotation matching through ChemSpider and mzCloud databases confirmed its identity with high confidence. This dual-mode detection highlights oleanolic acid as a prominent compound in DRF. Molecular docking results further revealed that oleanolic acid exhibited the strongest binding affinity towards AKT1 (− 10.2 kcal/mol) among the six tested compounds. Specifically, it formed a stable hydrogen bond with residue ALA283 in the AKT1 binding pocket.

To quantify oleanolic acid in DRF, HPLC analysis was performed using a standard curve generated from five concentrations (3.125–50 μg/mL), showing excellent linearity (R^2^ > 0.999) (Table S12 and Fig. S3). The retention time remained consistent across standards, with no interfering peaks. DRF extract (5 g sachet) was prepared as described and analyzed under identical conditions. The sample yielded a peak area of 414,100 mAU s, corresponding to 2.945 μg/mL. This value translates to 5.889 μg of OA in 5 g of extract, equating to 0.1178 μg/mg. Based on the dilution factor and experimental volumes, this corresponds to a final OA concentration of approximately 6.52 μM, which falls within the range (2.5–10 μM) used in our subsequent in vitro and in vivo experiments. These findings confirm that the experimental dosing is pharmacologically relevant and well-justified (Fig. S4).

To evaluate the protective effect of oleanolic acid (OA) against ethanol-induced hepatotoxicity, both in vitro and zebrafish models were employed. As shown in Fig. 6B, AML12 cells were exposed to increasing concentrations of ethanol (1.25%–10%) for 24 h. Cell viability remained unaffected at 1.25% ethanol, showing no significant difference compared to the control group (CTRL), with mean viability values of 97.96% ± 1.09% vs. 100.00% ± 5.79%, respectively. However, exposure to 5% and 10% ethanol dramatically reduced cell viability to below 50%, indicating substantial cytotoxicity and massive cell death. Notably, 2.5% ethanol exposure resulted in a moderate but significant reduction in cell viability (mean 62.85% ± 1.43%), suggesting partial hepatocellular damage while maintaining measurable viability for subsequent intervention. These findings confirm 2.5% ethanol as an appropriate and reliable concentration for ALD model establishment in vitro. Subsequent treatment with OA significantly restored the viability of ethanol-injured AML12 cells in a dose-dependent manner, comparable to the effect of dexamethasone (DXM) (Fig. 6C).

Based on previously established protocols [52], the zebrafish ALD model was constructed through ethanol exposure, which significantly reduced larval heart rate compared to the control group. Treatment with medium and high doses of OA significantly reversed this reduction, with effects like those of DXM (Fig. 6D). The experimental workflow and treatment timeline are illustrated in Fig. 6E. Phenotypic analysis further demonstrated that ethanol exposure caused a marked increase in morphological abnormalities, including swim bladder defects and signs of edema or body curvature. OA treatment notably reduced the incidence of moderate and severe phenotypes, particularly at higher doses (Fig. 6F). Representative larval images from each group are shown in Figs. 6G and S6, visually confirming the ameliorative effects of OA on ethanol-induced morphological damage. To further investigate the molecular interaction underlying these effects, CETSA was performed in AML12 cells. OA treatment significantly enhanced the thermal stability of AKT1 protein compared to DMSO, especially at 56 °C (*p* < 0.01), suggesting a direct binding interaction between OA and AKT1 (Fig. 6H and I). Collectively, these findings indicate that oleanolic acid exerts protective effects against ALD at both cellular and organismal levels, supporting its therapeutic potential for ALD.

## Discussion

The growing recognition of ALD as a major public health concern has highlighted the need for preventive strategies that can be easily incorporated into daily life. DRF is a traditional Chinese herbal tea developed by the National Master of Traditional Chinese Medicine, Professor Wang Qi. It has been widely used in clinical practice for improving liver function and regulating internal dampness-heat [51]. The growing recognition of ALD as a major public health concern has highlighted the need for preventive strategies that can be easily incorporated into daily life. Our study provides systematic evidence supporting the traditional use of DRF as a dietary-herbal approach for alcohol management and hepatic protection, offering a scientifically validated option for individuals seeking to mitigate alcohol-induced liver damage while maintaining social drinking habits. By integrating high-throughput data mining with advanced computational approaches, we elucidated the molecular basis of DRF’s therapeutic potential and identified AKT1 as a key molecular target contributing to its hepatoprotective effects.

Functional enrichment and protein interaction analyses revealed that DRF primarily modulates two pivotal ALD-related pathological processes: dysregulated lipid metabolism and chronic inflammation. KEGG pathway analysis highlighted significant enrichment in ALD- and metabolic-related signaling pathways, while GO annotations emphasized regulatory effects on enzymes such as alcohol dehydrogenases [52] and retinol dehydrogenases—critical mediators in ethanol metabolism [53]. In parallel, PPI network analysis identified AKT1 as a central hub, interfacing multiple disease-related pathways. These findings collectively suggest that DRF exerts multi-level regulatory effects on both ethanol detoxification and downstream inflammatory responses, thereby offering a comprehensive protective mechanism against alcohol-induced hepatic damage.

To robustly validate our target predictions, we conducted transcriptome-based differential gene expression analyses and implemented three machine learning algorithms (Random Forest, Support Vector Machine, and LASSO). Despite the limited sample size (n = 12), Random Forest achieved perfect classification (AUC = 1.0), with AKT1 consistently ranked as a top feature, indicating its strong association with ALD pathophysiology. SVM analysis, enhanced with a leave-one-out cross-validation (LOOCV) strategy to minimize overfitting, yielded an AUC of 0.965—far exceeding the baseline performance of conventional classifiers and underscoring the robustness of our core gene set. Integrative analysis identified AKT1, ICAM1, and HIF1A as shared features across all three models, yet AKT1 demonstrated superior performance across evaluation metrics, highlighting its prominence among the candidates. The consistent high ranking of AKT1 across algorithms with differing mathematical principles, together with its most favorable binding affinity and stability in molecular docking studies, strongly supports its central biological role for ALD. These observations align with existing literature, which implicates AKT1 as a major node in pathways related to inflammation [54], oxidative stress, and hepatic lipid accumulation [55, 56].

In contrast to conventional approaches that prioritize high-abundance compounds or predefined active ingredients, our study adopts a compound-centric strategy, treating the entire DRF formula as a multi-component small-molecule library. This design enables systematic and unbiased screening of hundreds of natural compounds—regardless of their relative abundance—against key protein targets implicated in ALD. Supported by high-performance computational resources, we conducted large-scale virtual screening of 690 compounds across five core targets, resulting in 3,450 compound–target docking events. This strategy not only enhances the comprehensiveness of target identification but also reflects a core advantage of traditional Chinese formulas—namely, their intrinsic structural diversity and multi-target potential. Compounds with low abundance but high binding affinity or favorable ADMET properties may serve as promising lead candidates for further development. By leveraging the formula as a natural compound reservoir, we were able to identify both well-known bioactives and potentially overlooked molecules with strong target engagement profiles [57]. This integrative framework aligns with the principles of network pharmacology and drug repurposing and may serve as a scalable model for future TCM-based discovery pipelines.

Our molecular docking analysis further confirmed the importance of AKT1, with multiple DRF-derived compounds demonstrating strong binding affinities (binding energy < − 8 kcal/mol) to the AKT1 protein. Among them, BH048 (oleanolic acid) exhibited the highest affinity (− 10.2 kcal/mol) and formed a stable hydrogen bond with residue ALA283, while BH060 interacted with THR341 and GLY394. These interactions highlight the potential of DRF to modulate AKT1 signaling, a pathway integral to hepatocyte survival and metabolic regulation. Additionally, the clustering of active compounds—mainly prenol lipids, flavonoids, and organooxygen compounds—suggests that the formula’s therapeutic effects stem from synergistic interactions among multiple structurally diverse components.

Comprehensive ADMET profiling provided further support for the drug-likeness of the key compounds, with favorable pharmacokinetic properties and minimal predicted hepatotoxicity or mutagenic risks. BH048, identified as oleanolic acid, displayed the most promising profile, exhibiting high binding affinity, low hepatotoxicity index (0.4139), and absence of mutagenicity. Given its confirmed presence in DRF and established pharmacological properties—ranging from lipid-lowering to anti-inflammatory effects oleanolic acid is likely a major contributor to DRF’s therapeutic efficacy. As a pentacyclic triterpenoid, oleanolic acid has been extensively documented for its beneficial effects in metabolic disorders, including diabetes, obesity, and hepatic steatosis, through mechanisms involving anti-inflammatory activity [58]. Although other compounds such as saucernetindiol and ursolic acid were not detected in untargeted metabolomics—likely due to low abundance or extraction limitations—they may still play roles in the overall pharmacological synergy of the formula.

To validate the functional relevance of oleanolic acid, we conducted a series of in vitro and in vivo experiments. In AML12 hepatocytes, oleanolic acid treatment significantly restored cell viability impaired by ethanol exposure, supporting its protective role against alcohol-induced cytotoxicity. Furthermore, in a zebrafish model of acute alcoholic liver injury, oleanolic acid improved heart rate, reduced morphological abnormalities (e.g., swim bladder non-inflation, edema), and partially rescued behavioral phenotypes. These results confirm its anti-ALD potential, though the precise molecular pathways involved remain to be clarified. Notably, the ADMET evaluation of oleanolic acid revealed favorable pharmacokinetic and toxicity profiles, with low predicted hepatotoxicity and mutagenicity risks, as well as high oral bioavailability potential. These features make it a strong candidate for further development and mechanistic investigation. Other compounds, such as BH060 and JYH072, also demonstrated strong docking scores, suggesting potential combinatorial effects within DRF.

To address concerns regarding the relevance of experimental dosing to real-world use, we quantified the oleanolic acid content in DRF and confirmed its presence at physiologically meaningful levels. Given that the adult daily dose involves the consumption of two 5 g sachets, the estimated total intake of oleanolic acid would proportionally increase, providing a plausible basis for systemic exposure. While factors such as intestinal absorption, hepatic metabolism, and compound bioavailability may influence the final circulating concentration, the range used in our in vitro and in vivo assays (2.5–10 μM) remains within the expected pharmacokinetic window. In our zebrafish model, oleanolic acid was administered by dissolving it directly into the fish water, with DMSO maintained at 0.1% (1/1000) to avoid solvent-induced toxicity, thereby ensuring safe and effective compound exposure. These findings support the translational validity of our experimental design and highlight the potential of DRF as a functionally relevant herbal formulation for hepatoprotection. It is important to recognize that although oleanolic acid is known for its poor aqueous solubility and consequently limited oral bioavailability, traditional multi-component formulations like DRF may offset this through synergistic matrix effects and formulation components. Indeed, various studies have demonstrated that combining oleanolic acid with natural excipients or herbal partners can improve its solubility, absorption, and metabolic stability [59]. These matrix-enhanced effects could underlie the robust hepatoprotective activity we observed, even at doses consistent with real-world intake. Such evidence supports considering DRF as an integrated delivery system, where the whole formulation—not just oleanolic acid alone—contributes to its therapeutic efficacy. Future work could explore these synergistic mechanisms in greater depth, potentially guiding formulation optimization and translational development.

Taken together, our study highlights a possible AKT1-centered mechanism through which DRF may exert therapeutic effects against ALD, with oleanolic acid emerging as a key bioactive component supported by both in silico prediction and experimental evidence. Notably, the CETSA confirmed the direct interaction between oleanolic acid and AKT1, suggesting potential target engagement at the protein level. However, while CETSA supports binding, no direct evidence currently demonstrates that oleanolic acid modulates AKT1 phosphorylation status or activates canonical AKT1-mediated signaling cascades. Classical downstream pathways of AKT1, such as the PI3K/AKT/mTOR or AKT/GSK3β axes, are well known to mediate metabolic regulation and cell survival, yet whether oleanolic acid acts through these canonical routes or engages alternative mechanisms remains to be elucidated. At present, the downstream consequences of this interaction are unclear, and no definitive mechanistic link has been established. Future studies will include phospho-specific Western blotting and kinase assays to assess changes in AKT1 phosphorylation and its downstream effectors, alongside integrated transcriptomic and proteomic analyses in animal models treated with oleanolic acid. These approaches will help delineate whether the observed therapeutic effects are mediated through classical AKT1 signaling or involve non-canonical pathways.

From a translational and integrative medicine perspective, our findings support the therapeutic promise of DRF as a traditional medicine-food homology formula with scientific evidence of efficacy against ALD. The identification of oleanolic acid as a key compound not only facilitates quality control and mechanistic standardization but also bridges TCM theory with modern biomedical research. These results align with the holistic and multi-target principles of TCM, emphasizing the synergy of compound formulas rather than isolated ingredients. Further preclinical and clinical studies will be necessary to fully characterize the pharmacodynamic properties and therapeutic mechanisms of DRF and its key constituents. This work contributes to the growing body of research that integrates traditional formulations with contemporary pharmacological strategies, and lays a foundation for the development of safe, effective, and culturally compatible interventions for  ALD.

## Conclusion

This study highlights the utility of combining transcriptomic analysis, machine learning, molecular docking, and pharmacological validation to uncover the active components and potential mechanisms of DRF—a traditional formula composed of nine medicine-food homology herbs. Among the predicted bioactive compounds, oleanolic acid was identified as a key candidate with favorable ADMET properties and demonstrated protective effects against ethanol-induced liver injury in both hepatocyte and zebrafish models. Although its direct regulatory relationship with AKT1 requires further investigation, these findings support the therapeutic potential of DRF as a multi-component, multi-target intervention for ALD. By aligning traditional wisdom with modern scientific validation, this work contributes to the ongoing modernization of Chinese medicine and supports the development of safe and effective dietary-pharmacological strategies for managing ALD.

## Supplementary Information

Supplementary material 1.

## Data Availability

The data that support the findings of this study are included in the paper and its supplementary information files.

## Abbreviations

ADMET: Absorption, distribution, metabolism, excretion, and toxicity
AKT1: RAC-α serine/threonine-protein kinase 1
ALD: Alcoholic liver disease
BATMAN-TCM: Bioinformatics Analysis Tool for Molecular mechanism of Traditional Chinese Medicine
BH: *Mentha* *haplocalyx* (Bo he)
BP: Biological process
CESTA: Cellular thermal shift assay
CTD: Comparative Toxicogenomics Database
CTRL: Control
CXD: *Phaseoli semen* (Chi xiao dou)
CXCL8: C-X-C motif chemokine ligand 8
DAVID: Database for Annotation, Visualization, and Integrated Discovery
DEGs: Differentially expressed genes
DMSO: Dimethyl sulfoxide
DRF: Dampness-Heat Regulating Formula
DXM: Dexamethasone
DZY: *Lophatheri* *herba* (Dan zhu ye)
FBS: Fetal bovine serum
GEO: Gene Expression Omnibus
GO: Gene Ontology
H-HT: Human hepatotoxicity
HIA: Human intestinal absorption
HPLC: High-performance liquid chromatography
HX: *Pogostemon* *cablin* (Huo xiang)
IL6: Interleukin-6
IPA: Isopropyl alcohol
ITS: Insulin-transferrin-selenium
JYH: *Lonicera* *japonica* (Jin yin hua)
KEGG: Kyoto Encyclopedia of Genes and Genomes
LASSO: Least Absolute Shrinkage and Selection Operator
LOOCV: Leave-One-Out Cross-Validation
MCL: Markov clustering
MCX: *Portulaca oleracea* (Ma chi xian)
MDS: Multidimensional scaling
MF: Molecular function
NAD ^+^: Nicotinamide adenine dinucleotide
NFKB1: Nuclear Factor Kappa B1
PAINS: Pan Assay Interference Compounds
PFA: 4% Paraformaldehyde fixative
PGY: *Taraxaci* *herba* (Pu gong ying)
PPB: Plasma Protein Binding
PPI: Protein–Protein Interaction
RBF: Radial basis function
RDK: *Myristica* *fragrans* (Rou dou kou)
RF: Random Forest
ROA: Route of Administration
SVM: Support Vector Machine
TCM: Traditional Chinese Medicine
TCMSP: Traditional Chinese Medicine Systems Pharmacology Database and Analysis Platform
TLR4: Toll-like receptor 4
TNF: Tumor Necrosis Factor
VST: Variance stabilizing transformation
YR: *Coicis* *semen* (Yi yi ren)
