# Supplementary material for: Discovery of an AKT1-targeting compound from a traditional herbal formula for alcoholic liver disease via integrative computational and experimental approaches
Source: Chin Med. 2025 Oct 7;20:166. doi: 10.1186/s13020-025-01205-y (PMC12502445; doi:10.1186/s13020-025-01205-y)
Supplement: Supplementary file 1 — Supplementary material 1. [file 13020_2025_1205_MOESM1_ESM.zip › Yang et al_Supplementary material/Yang et al_Supplementary Materials_5Sep2025.docx]

Supplementary Materials for

Discovery of an AKT1-targeting compound from a traditional herbal formula for alcoholic liver disease via integrative computational and experimental approaches

*Shuxuan Yang^a,1^, Caiting Zou^a,1^, Dexian Li^a,1^, Jingxin Lin^a^, Qinghong Chen^a^，Meilin Chen^a^, Chuanghai Wu^a^, Andrew Hung^b^, Yanyan Liu^a^, Xiaomin Sun^a^, Hong Li^a,^**^b,*^, Qi Wang^c^, Xiaoshan Zhao^a,d,*^*

**Contents**

[Table S1. Characteristics of the from compounds identified from Dampness-heat Regulating Formula. 3](#_Toc1790732115)

[Table S2. Details of Kyoto Encyclopedia of Genes and Genomes Pathway enrichment analysis. 4](#_Toc1998827747)

[Table S3. Node degree of genes from alcoholic liver disease pathway. 5](#_Toc1211253808)

[Table S4. Details of Gene Ontology enrichment analysis. 6](#_Toc1545261143)

[Table S5. Raw data of ligand molecular docking results to five targets individually. 7](#_Toc1684287230)

[Table S6. Mass spectrometry raw data of Dampness-heat Regulating Formula granules. 8](#_Toc1833523503)

[Table S7. Details of differential expressed genes. 9](#_Toc1786664118)

[Table S8. Top 15 genes ranked by importance in the random forest model. 10](#_Toc199995225)

[Table S9. Top 10 genes identified by the support vector machine model. 11](#_Toc507839020)

[Table S10. Sample-level prediction probability output from the LASSO classification model 12](#_Toc1150395962)

[Table S11. Key gene coefficients identified by LASSO regression 13](#_Toc909659393)

[Table S12. Calibration data of oleanolic acid (OA) for HPLC quantification. 14](#_Toc709335158)

[Fig. S1. The cluster network of each herb in the Dampness-heat Regulating Formula based on structural similarity. 15](#_Toc1114276009)

[Fig. S2. Principal component analysis reveals distinct clustering of ALD and control samples. 18](#_Toc1579481423)

[Figure S3. Calibration curve of OA standard constructed from HPLC data. 19](#_Toc1298915794)

[Figure S4. HPLC chromatograms of oleanolic acid (OA) standard and DRF extract. 20](#_Toc1706478003)

[Figure S5. Replicate CETSA blots confirming OA–AKT1 interaction. 21](#_Toc1131690736)

[Figure S6. Oil Red O staining of 4.5 days post-fertilization zebrafish larvae to assess hepatic lipid accumulation. 22](#_Toc63538473)

[Information S1. ARRIVE 2.0 compliance checklist for reporting](#_Toc591743152) *[in vivo](#_Toc591743152)* [experiments. 23](#_Toc591743152)

[Information S2. Dataset description and processing pipeline. 24](#_Toc430386407)

[Information S3. Reagent Specifications. 25](#_Toc779419353)

**Table S1. Characteristics of the from compounds identified from Dampness-heat Regulating Formula.**

The content is in the document “Supplementary Table S1. Characteristics of the from compounds identified from Dampness-heat Regulating Formula.xlsx”.

**Note**: N/A, not appliable; AlogP, Partition coefficient between octanol and water; BBB, Blood-brain barrier; Caco-2, Human intestinal cell line Caco-2 permeability; DL, Drug-likeness; FASA-, Fractional water accessible surface area of all atoms with negative partial charge; Hacc, Hydrogen-bond acceptors; H-bond, Hydrogen-bond donors; HL, Drug half-life; MW, Molecular weight; OB, Oral bioavailability. All data was retrieved from the Traditional Chinese Medicine Systems Pharmacology Database and Analysis Platform (<https://tcmsp-e.com/>).

**Table S2. Details of Kyoto Encyclopedia of Genes and Genomes Pathway enrichment analysis.**

| Description | Gene ratio | *p* value | Gene ID | Count |
| --- | --- | --- | --- | --- |
| Lipid and atherosclerosis | 0.42 | 1.08E-13 | CXCL8/ STAT3/ MAPK14/ SELE/ TNF/ MMP9/ NFKB1/ IL6/ CASP1/ CYP1A1/ AKT1/ PPARG/ TLR4/ NFE2L2 | 14 |
| Alcoholic liver disease | 0.36 | 9.35E-13 | IL6/ CXCL8/ ALDH2/ ADH1C/ ADH1B/ ADH1A/ AKT1/ MAPK14/ PPARA/ TNF/ TLR4/ NFKB1 | 12 |
| Chagas disease | 0.27 | 1.63E-09 | IL6/ ACE/ CXCL8/ AKT1/ MAPK14/ TNF/ TLR4/ IL2/ NFKB1 | 9 |
| Yersinia infection | 0.27 | 1.68E-08 | IL6/ CXCL8/ CASP1/ AKT1/ MAPK14/ TNF/ TLR4/ IL2/ NFKB1 | 9 |
| AGE-RAGE signaling pathway in diabetic complications | 0.24 | 4.33E-08 | IL6/ CXCL8/ STAT3/ AKT1/ MAPK14/ SELE/ TNF/ NFKB1 | 8 |
| Hepatitis B | 0.27 | 6.19E-08 | IL6/ CXCL8/ STAT3/ AKT1/ MAPK14/ TNF/ TLR4/ MMP9/ NFKB1 | 9 |
| Coronavirus disease - COVID-19 | 0.30 | 7.68E-08 | IL6/ ACE/ CXCL8/ STAT3/ CASP1/ MAPK14/ TNF/ TLR4/ IL2/ NFKB1 | 10 |
| Pertussis | 0.21 | 2.40E-07 | IL6/ CXCL8/ CASP1/ MAPK14/ TNF/ TLR4/ NFKB1 | 7 |
| Fluid shear stress and atherosclerosis | 0.24 | 4.32E-07 | GSTP1/ AKT1/ MAPK14/ SELE/ TNF/ MMP9/ NFKB1/ NFE2L2 | 8 |
| Non-alcoholic fatty liver disease | 0.24 | 8.96E-07 | IL6/ CXCL8/ AKT1/ PPARG/ MAPK14/ PPARA/ TNF/ NFKB1 | 8 |

**Table S3. Node degree of genes from alcoholic liver disease pathway.**

| Node | Node identifier | Node degree |
| --- | --- | --- |
| ADH1A | 9606.ENSP00000209668 | 3 |
| ADH1B | 9606.ENSP00000306606 | 3 |
| ADH1C | 9606.ENSP00000426083 | 3 |
| AKT1 | 9606.ENSP00000451828 | 6 |
| ALDH2 | 9606.ENSP00000261733 | 3 |
| CXCL8 | 9606.ENSP00000306512 | 6 |
| IL6 | 9606.ENSP00000385675 | 6 |
| MAPK14 | 9606.ENSP00000229795 | 5 |
| PPARA | 9606.ENSP00000385523 | 5 |
| TLR4 | 9606.ENSP00000363089 | 6 |
| TNF | 9606.ENSP00000398698 | 6 |

**Table S4. Details of Gene Ontology enrichment analysis.**

| **GO term** | **-log10(*p*)** | **Count** | **Subgroup** |
| --- | --- | --- | --- |
| Positive regulation of gene expression | 9.622150056 | 12 | Biological process |
| Positive regulation of transcription by RNA polymerase II | 8.575888637 | 15 |  |
| Positive regulation of DNA-templated transcription | 8.087087472 | 12 |  |
| Regulation of transcription by RNA polymerase II | 1.890233556 | 8 |  |
| Chromatin | 3.561703499 | 9 | Cellular component |
| Nucleoplasm | 3.431302264 | 16 |  |
| Nucleus | 0.474137597 | 12 |  |
| Alcohol dehydrogenase (NAD^+^) activity, zinc-dependent | 4.254791907 | 3 | Molecular function |
| Alcohol dehydrogenase (NAD^+^) activity | 3.925148649 | 3 |  |
| All-trans-retinol dehydrogenase (NAD^+^) activity | 3.304104759 | 3 |  |

**Table S5. Raw data of ligand molecular docking results to five targets individually.**

The content is in the document “Supplementary Table S5. Raw data of ligand molecular docking results to 5 targets individually.xlsx”.

**Table S6. Mass spectrometry raw data of Dampness-heat Regulating Formula granules.**

The content is in the document “Supplementary Table S6. Mass spectrometry raw data of Dampness-heat Regulating Formula granules.xlsx”.

**Note:** DeltaMass, the difference between measured and calculated mass in parts per million (ppm); Calc. MW, Calculated Molecular Weight; m/z, mass-to-charge ratio; RT: Retention Time, measured in minutes; Area (Max.), peak area representing the maximum intensity

**Table S7. Details of differential expressed genes.**

The content is in the document “Supplementary Table S7. Details of differential expressed genes.xlsx”.

Note: baseMean, the average normalized expression of the gene across all samples; log2FoldC, log2-transformed fold change between ALD vs. CTRL; padj, p-value adjusted for multiple testing using the Benjamini-Hochberg method (false discovery rate); significant, indicates whether the gene meets the significance threshold; lfcSE, standard error of the log2 fold change estimate; Stat, test statistic value used to calculate the p-value.

**Table S8. Top 15 genes ranked by importance in the random forest model.**

This table presents the top-ranked genes selected by the random forest algorithm based on their importance scores in distinguishing alcoholic liver disease from healthy controls. AKT1, CXCL8, and AKR1B1 were among the highest-ranked features, suggesting their potential involvement in disease-related pathways. Negative importance indicates a minor inverse association in the model.

| Gene | Importance |
| --- | --- |
| AKT1 | 0.0695 |
| CXCL8 | 0.048833333 |
| AKR1B1 | 0.044333333 |
| THBS2 | 0.044 |
| ICAM1 | 0.040666667 |
| HIF1A | 0.038166667 |
| MMP14 | 0.037666667 |
| BCL2L1.1 | 0.019666667 |
| APPAT | 0.017833333 |
| F2R | 0.014666667 |
| MMP2 | 0.012333333 |
| SELENON | 0.011166667 |
| BCL2L1 | 0.010166667 |
| PPARD | 0.0055 |
| TYROBP | -0.002 |

**Table S9. Top 10 genes identified by the support vector machine model.**

This table shows the top 10 genes ranked by importance scores derived from the support vector machine algorithm. A score of 1 indicates maximum contribution to classification accuracy. Genes such as CXCL8, AKR1B1, and AKT1 demonstrated the highest discriminative power in distinguishing alcoholic liver disease samples from healthy controls. Repeated entries (e.g., BCL2L1 and BCL2L1.1) reflect transcript variants with similar predictive importance.

| **Gene** | **Importance** |
| --- | --- |
| CXCL8 | 1 |
| AKR1B1 | 1 |
| AKT1 | 1 |
| ICAM1 | 1 |
| MMP14 | 1 |
| THBS2 | 1 |
| HIF1A | 1 |
| BCL2L1 | 0.942857142857143 |
| BCL2L1.1 | 0.942857142857143 |
| F2R | 0.942857142857143 |

**Table S10. Sample-level prediction probability output from the LASSO classification model.**

This table displays the predicted probabilities for each sample based on the LASSO regression model. Positive values correspond to classification toward the alcoholic liver disease (ALD) group, while negative values indicate classification toward the control group. All true labels are correctly assigned, confirming the model's robust performance under leave-one-out cross-validation.

| **Sample** | **True label** | **Predicted probability** |
| --- | --- | --- |
| ALD_1 | ALD | 2.48287154484947 |
| ALD_2 | ALD | 7.05241969057769 |
| ALD_3 | ALD | -1.642402221 |
| ALD_4 | ALD | 3.32040544280018 |
| ALD_5 | ALD | 13.887901495437 |
| ALD_6 | ALD | 2.64147266523627 |
| ALD_7 | ALD | 9.58317061766238 |
| CTRL_1 | CTRL | -3.700279401 |
| CTRL_2 | CTRL | -2.375800243 |
| CTRL_3 | CTRL | -1.397822173 |
| CTRL_4 | CTRL | -4.576254814 |
| CTRL_5 | CTRL | -2.93865962 |

**Table S11. Key gene coefficients identified by LASSO regression.**

This table summarizes the coefficients of genes retained in the final LASSO model. Genes with non-zero coefficients were considered predictive features. AKT1 exhibited the highest coefficient value, suggesting its primary contribution to the model’s classification power. The absolute coefficient values indicate relative feature importance across the selected predictors.

| **Gene** | **Coefficient** | **Absolute Coefficient** |
| --- | --- | --- |
| AKT1 | 1.902101947 | 1.902101947 |
| ICAM1 | 0.244851593 | 0.244851593 |
| HIF1A | 0.118994056 | 0.118994056 |

**Table S12. Calibration data of oleanolic acid (OA) for HPLC quantification.**

**A series of OA standard solutions at concentrations ranging from 3.125 to 50 μg/mL were prepared and analyzed using HPLC. The corresponding peak areas (mAU·s) were recorded to construct the calibration curve used for quantifying OA in DRF samples.**

| **Standards** | **Concertration(μg/mL)** | **Peak area (mAU·S)** |
| --- | --- | --- |
| 1 | 3.125 | 427210 |
| 2 | 6.25 | 743598 |
| 3 | 12.5 | 1609057 |
| 4 | 25 | 3022422 |
| 5 | 50 | 5934324 |

**Fig. S1. The cluster network of each herb in the Dampness-heat Regulating Formula based on structural similarity.**

Figs. S1A–I show cluster networks of compounds from JYH, BH, HX, CXD, MCX, RDK, YYR, PGY, and DZY, respectively. Nodes represent DRF compounds; edges indicate structural similarity. Node color reflects binding affinity to AKT1, and node size reflects a combination of binding strength and similarity—larger nodes indicate lower affinity and greater similarity to neighbors.


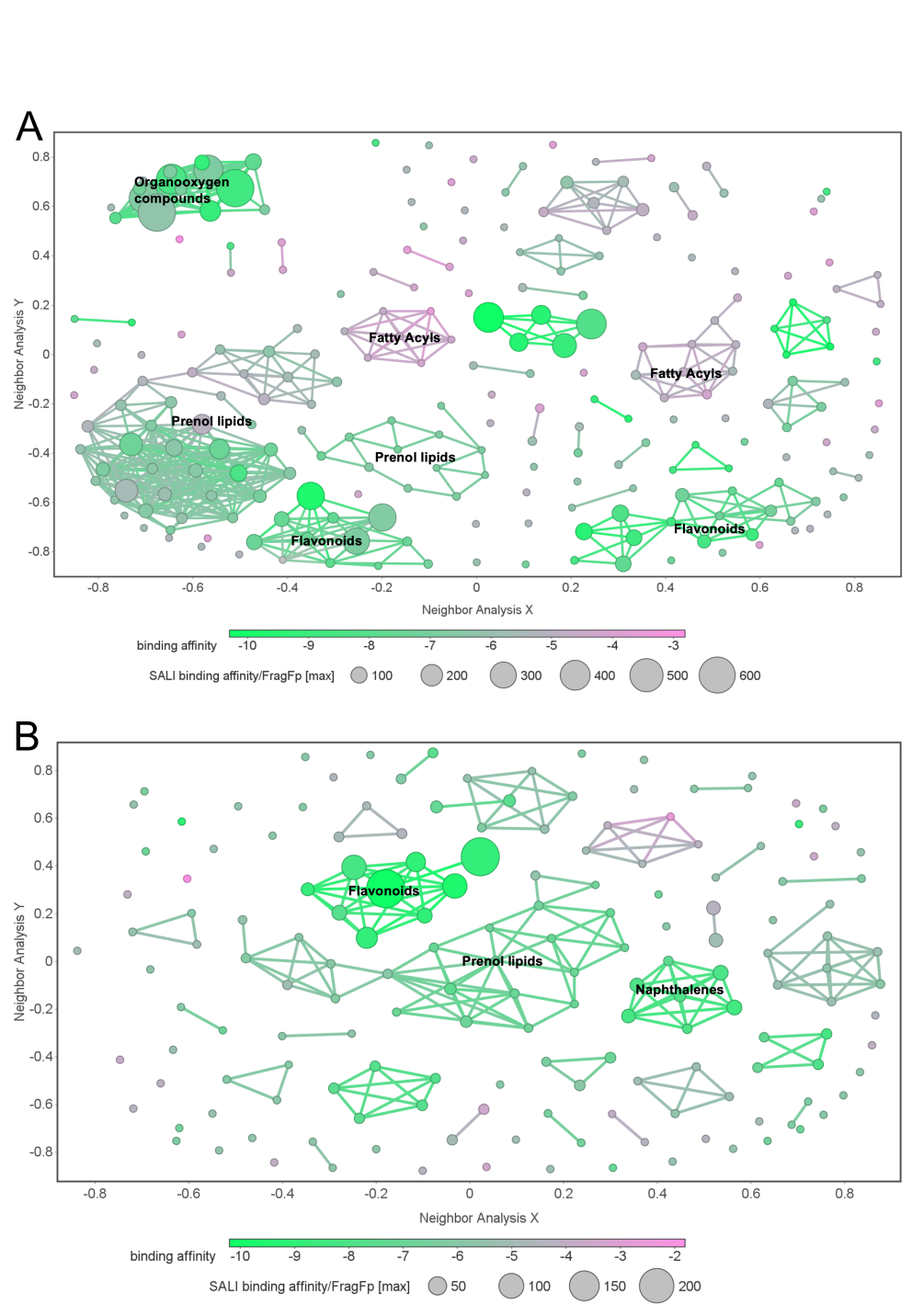

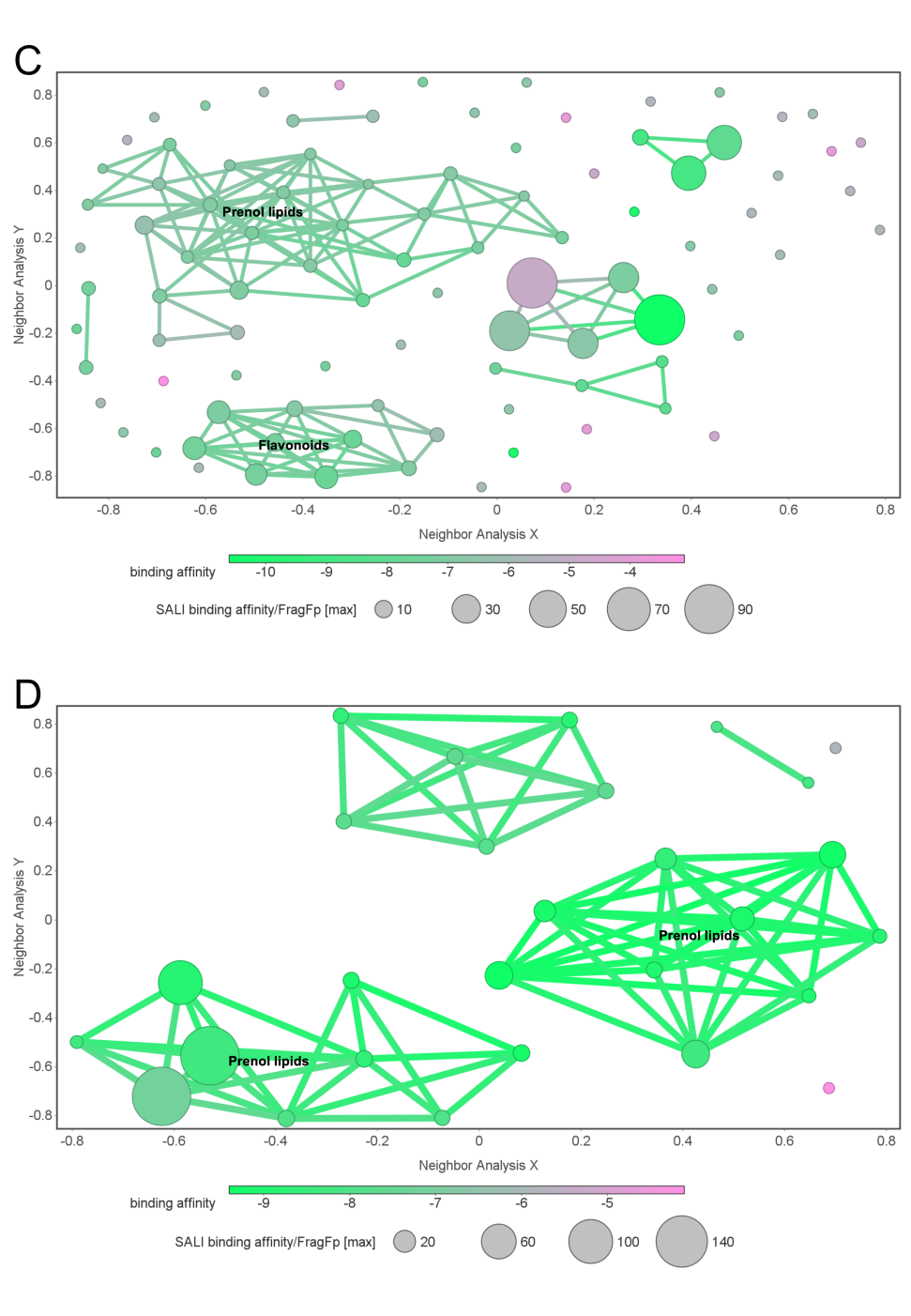


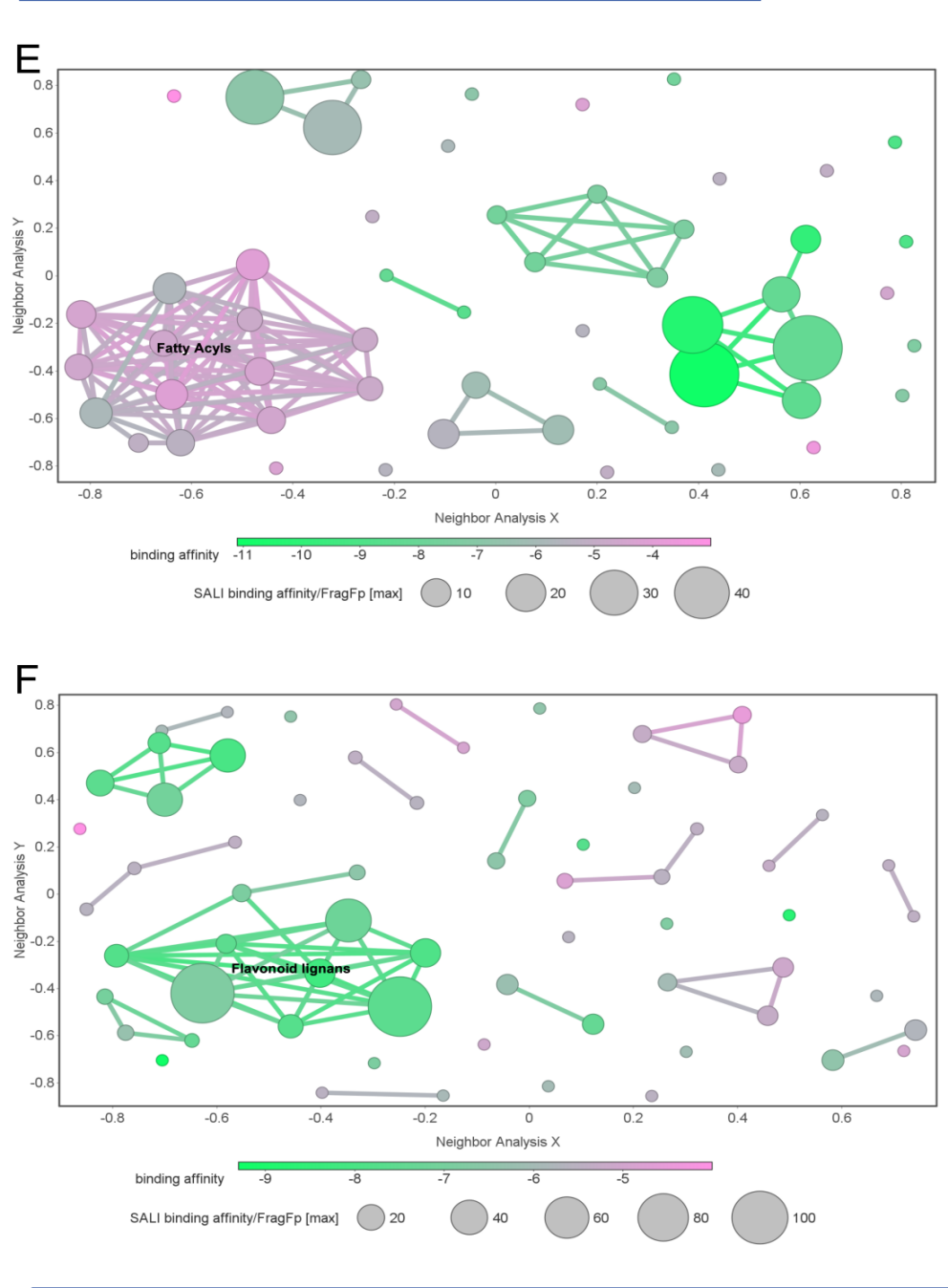

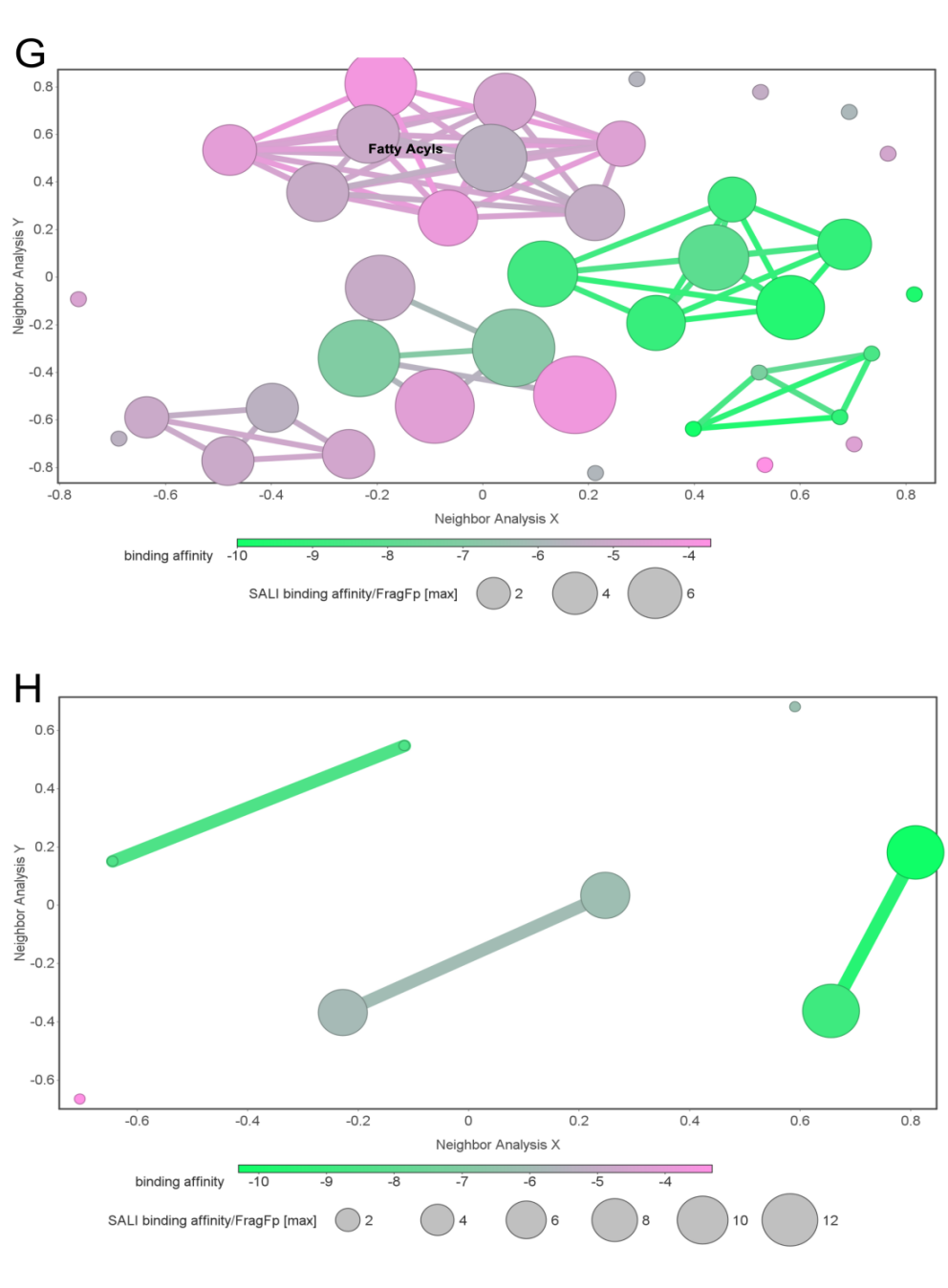


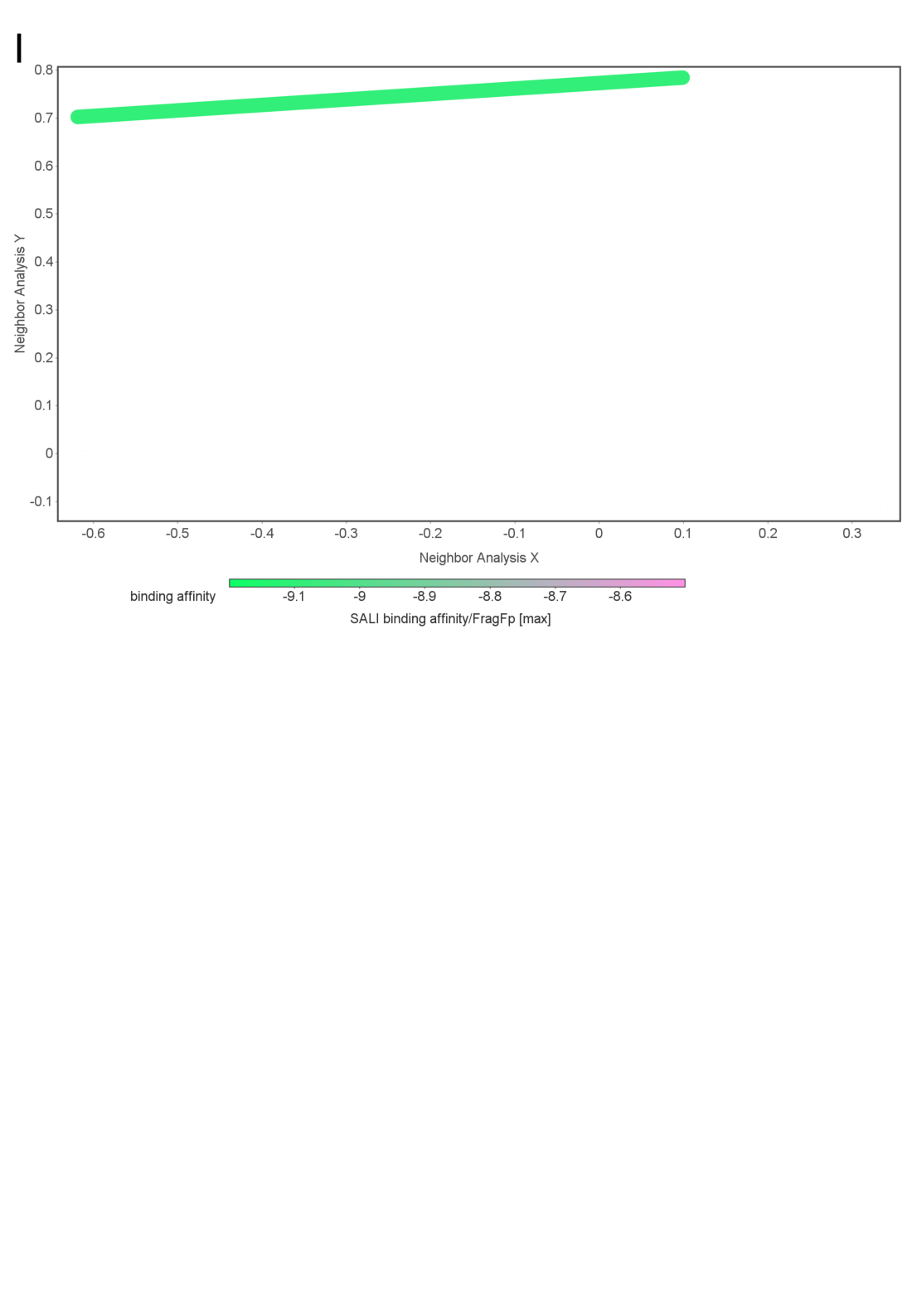


**Fig. S2. Principal component analysis reveals distinct clustering of ALD and control samples.**

**
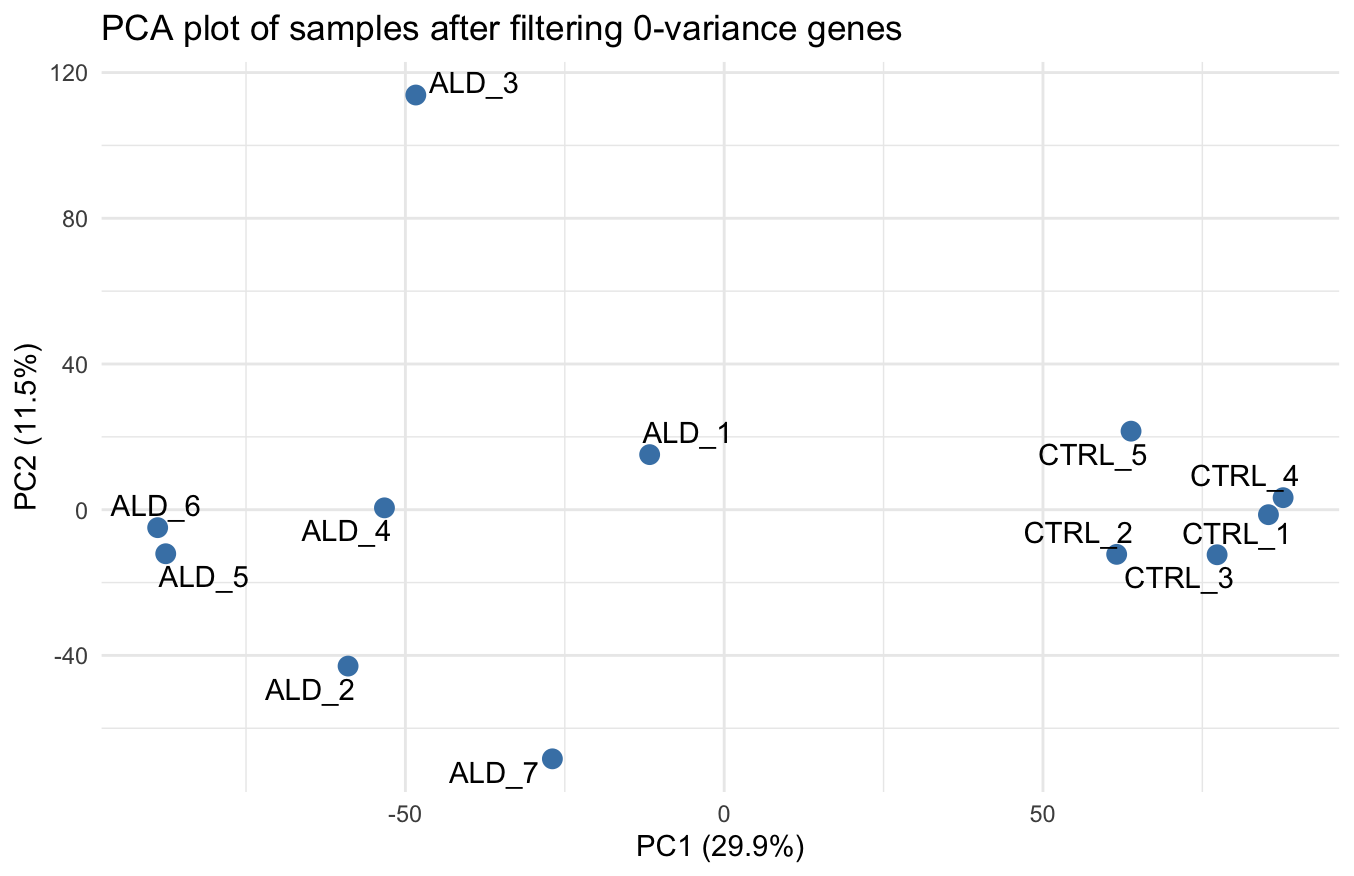
**

**Figure S3. Calibration curve of OA standard constructed from HPLC data.**

A standard curve was generated by plotting peak area (mAU·s) against OA concentration (μg/mL). The regression equation is y = 117660.66x + 67647, with a high coefficient of determination (R^2^ = 0.9996), indicating excellent linearity within the tested concentration range.

**
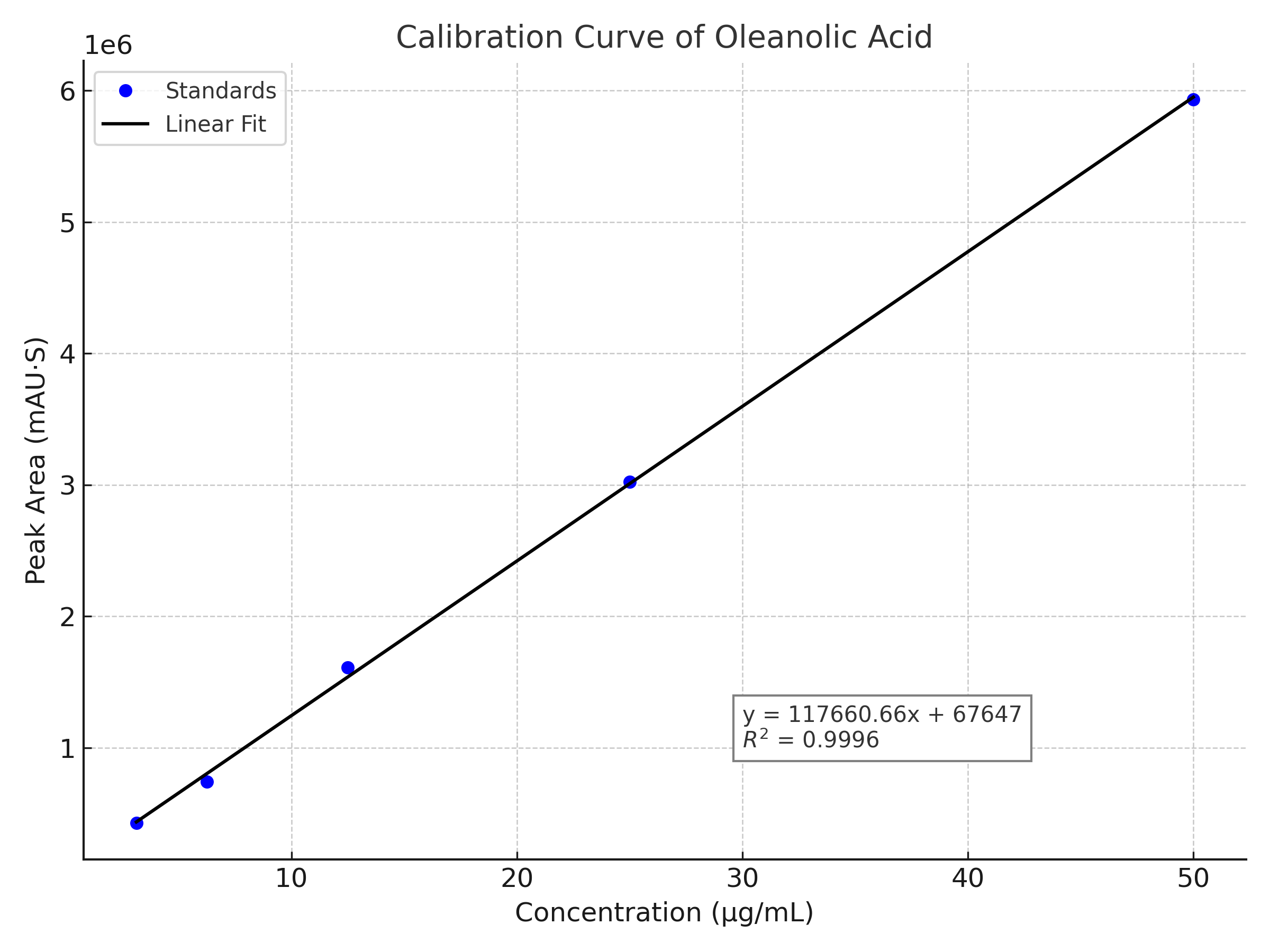
**

**Figure S4. HPLC chromatograms of oleanolic acid (OA) standard and DRF extract.**

Representative chromatograms of OA standard (6.25 μg/mL, top panel) and DRF extract (bottom panel) are shown. The DRF extract was prepared by dissolving 5 g of DRF in methanol, followed by ultrasonic-assisted extraction and rotary evaporation. Magnified views on the right highlight the retention time region corresponding to OA. The retention time and peak shape of OA in the sample matched that of the standard, confirming the presence of OA in DRF.

**
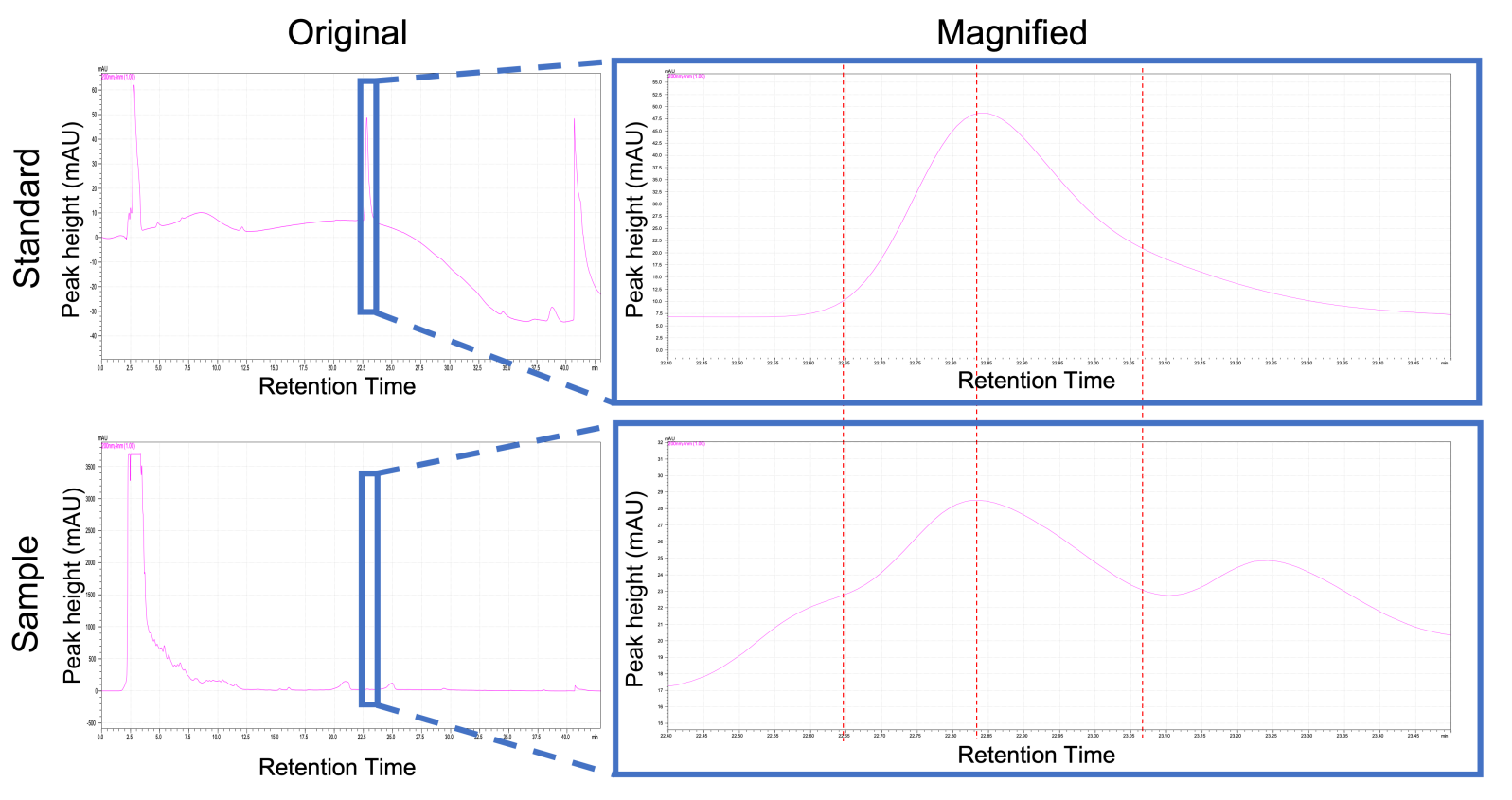
**

**Figure S5. Replicate CETSA blots confirming OA–AKT1 interaction.**

Three independent replicates of the Cellular Thermal Shift Assay (CETSA) conducted alongside the experiment shown in Figure 6H, using AML12 cell lysates treated with OA or DMSO. All replicates consistently demonstrated enhanced thermal stability of AKT1 in the OA group across the tested temperature range (40–70 °C), supporting the reproducibility of compound–target engagement.

**
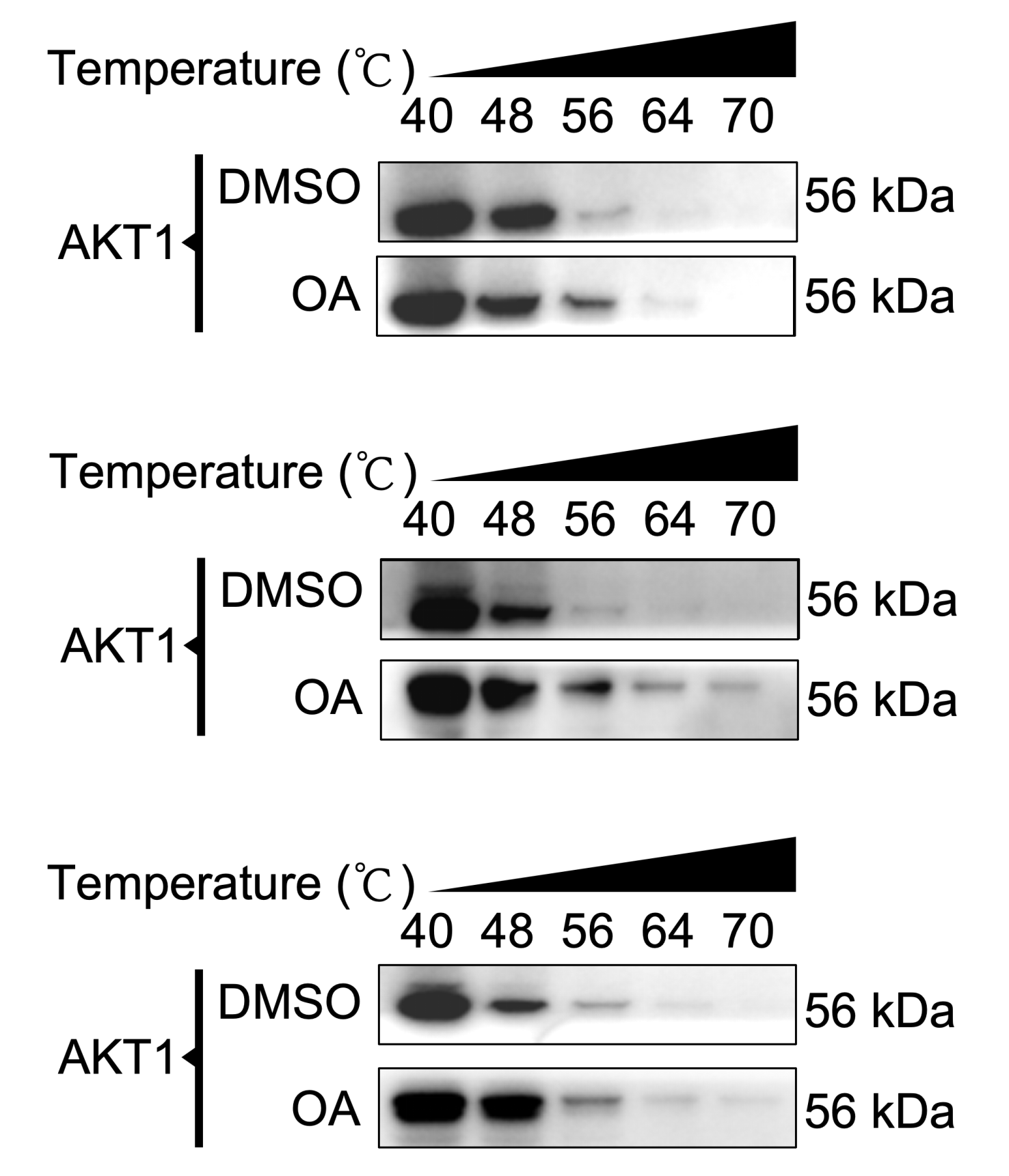
**

**Figure S6. Oil Red O staining of 4.5 days post-fertilization zebrafish larvae to assess hepatic lipid accumulation.**


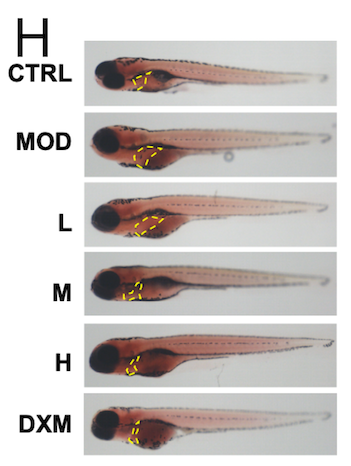
**
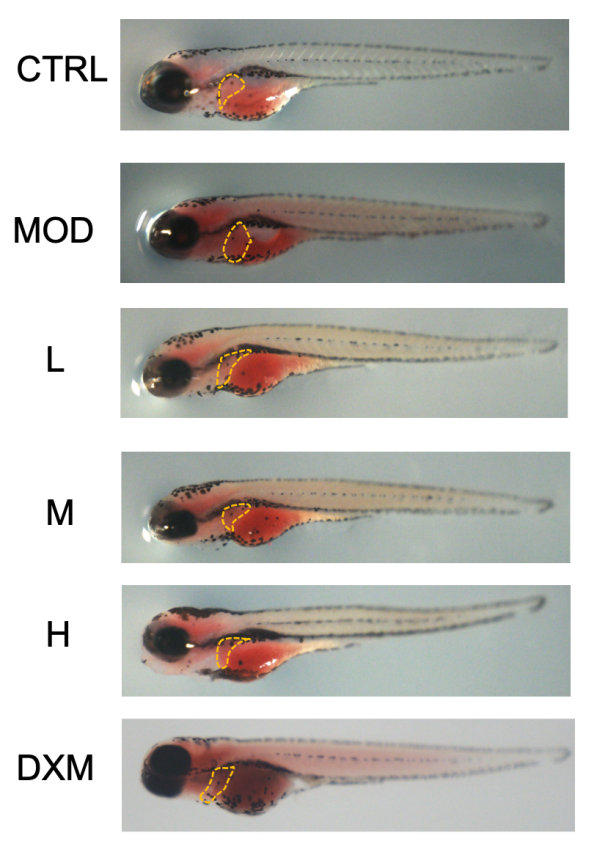
**

**Information S1. ARRIVE 2.0 compliance checklist for reporting *in vivo* experiments.**


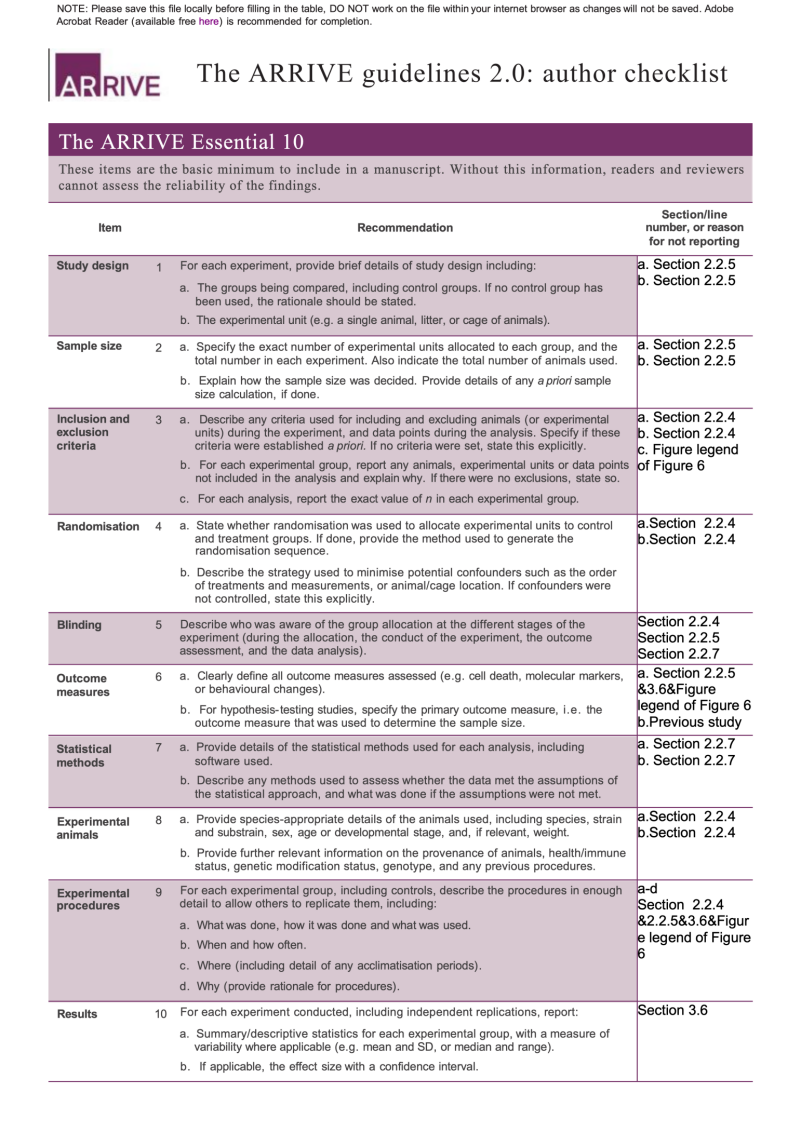


**Information S2. Dataset description and processing pipeline.**

**1.1 Dataset overview**

- GEO Accession: GSE167308
- Title: Pathogenesis of alcoholic hepatitis
- Platform: GPL20301 (Illumina HiSeq 4000)
- Organism: Homo sapiens
- Samples: 19 total (7 alcoholic hepatitis, 6 alcoholic cirrhosis, 6 controls)
- Submission Date: February 23, 2021
- Last Update: February 22, 2022
- PubMed Reference: PMID: 34129887
- Raw Data Availability: NCBI GEO ([https://www.ncbi.nlm.nih.gov/geo/query/acc.cgi?acc=GSE167308](https://www.ncbi.nlm.nih.gov/geo/query/acc.cgi?acc=GSE167308))

**1.2 Sample characteristics**

| **Group** | **Sample name / GEO Accession** | **Disease** |
| --- | --- | --- |
| ALD_1 | GSM5101243 | Alcoholic hepatitis |
| ALD_2 | GSM5101244 | Alcoholic hepatitis |
| ALD_3 | GSM5101245 | Alcoholic hepatitis |
| ALD_4 | GSM5101246 | Alcoholic hepatitis |
| ALD_5 | GSM5101247 | Alcoholic hepatitis |
| ALD_6 | GSM5101248 | Alcoholic hepatitis |
| ALD_7 | GSM5101249 | Alcoholic hepatitis |
| CTRL_1 | GSM5101257 | Control |
| CTRL_2 | GSM5101258 | Control |
| CTRL_3 | GSM5101259 | Control |
| CTRL_4 | GSM5101260 | Control |
| CTRL_5 | GSM5101261 | Control |

**1.3 Experimental Protocol**

- Tissue Processing: Microdissection of hepatocytes from frozen human liver biopsies
- RNA extraction: PicoPure RNA Extraction Kit (Excilone 0204)
- Library Preparation: cDNA synthesis: Ovation RNA-seq V2 from 5 ng total RNA
- Fragmentation: Covaris E220 sonication
- Library construction: Ovation Ultralow Library System V2 (6 PCR cycles)
- Sequencing: Illumina HiSeq 4000 platform
- Read format: 150 bp paired-end
- Base calling software: RTA 2.7.3 / bcl2fastq 2.17.1.14

**1.4 Bioinformatics Analysis**

Read alignment: STAR v2.5.3a (mapped to hg38 human reference genome)

Transcript quantification: HTSeq v0.6.1p1 using Ensembl release 96 annotations

Raw count matrix: Available in TSV format from GEO

**1.5 Quality Control Metrics**

All selected samples passed stringent QC criteria:

RNA integrity number (RIN) > 7

Library size > 20 million reads

Mapping rate > 85% (using STAR)

**Information S3. Reagent Specifications.**

Oleanolic acid (MedChemExpress, Shanghai, China; CAS: 508-02-1; Catalog: HY-N0156; Lot: 17668) was used for experimental treatments. Dexamethasone (Shanghai Yuanye Bio-Technology Co., Ltd., Shanghai, China; Catalog: S17003; Lot: S12HS194411) served as a positive control in pharmacological assays. Absolute ethanol (Tianjin Damao Chemical Reagent Co., Ltd., Tianjin, China; CAS: 64-17-5) was utilized for solvent preparation and cell model induction.

Cell culture was maintained in DMEM/F-12 (1:1) medium (Gibco, Thermo Fisher Scientific Inc., Waltham, MA, USA; Catalog: C11330500BT; Lot: 6124022) supplemented with antibiotics and growth factors. The antibiotic solution contained streptomycin/penicillin (Procell Life Science & Technology Co., Ltd., Wuhan, China; Catalog: PB180120), while insulin-transferrin-selenium (ITS) supplement (Beyotime Biotechnology Co., Ltd., Shanghai, China; Catalog: C0341-10ml; Lot: A033250107) provided essential growth factors.

Fetal bovine serum (Beijing Innochem Technology Co., Ltd., Beijing, China; Catalog: BFBSU1-500ML; Lot: KYIL7020) was heat-inactivated prior to use in cell culture. Dimethyl sulfoxide (DMSO; PYTHONBIO Co., Ltd., Nanjing, China; Catalog: D2650; CAS: 67-63-5) served as the vehicle control for compound solubilization. Cell viability was assessed using Cell Counting Kit-8 (CCK-8; Biosharp Life Sciences, Hefei, China; Catalog: BS350A).

Lipid accumulation was visualized using Oil Red O stain (Aladdin Biochemical, Shanghai, China; CAS: 1320-06-5; Catalog: L486491), with isopropyl alcohol (Tianjin Baishi Chemical Co., Ltd., Tianjin, China; CAS: 104-76-7) employed for dye extraction. Tissue fixation was performed with 4% paraformaldehyde (Biosharp Life Sciences, Hefei, China; Catalog: BL539A; Lot: 24171851) to preserve cellular morphology.
